# The nasal and oropharyngeal microbiomes of healthy livestock workers

**DOI:** 10.1371/journal.pone.0212949

**Published:** 2019-03-12

**Authors:** Ashley E. Kates, Mark Dalman, James C. Torner, Tara C. Smith

**Affiliations:** 1 Department of Epidemiology, College of Public Health, University of Iowa, Iowa City, IA, United States of America; 2 Kent State University, College of Public Health, Kent, OH, United States of America; Universitatsklinikum Munster, GERMANY

## Abstract

Little information exists on the microbiomes of livestock workers. A cross-sectional, epidemiological study was conducted enrolling 59 participants (26 of which had livestock contact) in Iowa. Participants were enrolled in one of four ways: from an existing prospective cohort study (n = 38), from the Iowa Department of Natural Resources Animal Feeding Operations database (n = 17), through Iowa county fairs (n = 3), and through snowball sampling (n = 1). We collected swabs from the nares and oropharynx of each participant to assess the microbiome via 16s rRNA sequencing. We observed livestock workers to have greater diversity in their microbiomes compared to those with no livestock contact. In the nares, there were 27 operational taxonomic units found to be different between livestock workers and non-livestock workers with the greatest difference seen with *Streptococcus* and *Proteobacteria*. In the oropharynx, livestock workers with swine exposure were more likely to carry several pathogenic organisms. The results of this study are the first to characterize the livestock worker nasal and oropharyngeal microbiomes.

## Introduction

The importance of microorganisms in maintaining human health has been recognized for many years. The composition of the microbiome is greatly influenced by one’s environment [1]. It has been hypothesized the composition of the microbiome may protect those raised on farms from developing diseases such as asthma and atopy through animal-associated microbes and plant materials that stimulate the immune system (the farm effect) [2].

Interestingly, this farm effect is most often attributed to modulating childhood development of the innate and humoral immune system of many of these immune dysfunction diseases [3]. Adults working in close proximity to animals are at increased risk of respiratory conditions including chronic obstructive pulmonary disease (COPD), occupational asthma, and organic dust toxic syndrome [4]. This is in part due to the inhalation of organic dust containing microorganisms [5, 6] that result in a dysregulation of the host immune response. This is especially true for individuals working in enclosed animal houses found commonly in swine and poultry production.

In order to better understand the relationship between the microbiome and livestock worker’s health, research is needed to characterize the microbiome of those with livestock contact. While research exists characterizing the environment around livestock production facilities as well as the livestock, there is surprising limited information on the workers themselves. To date, we are aware of only two studies assessing the nasal and oral microbiomes of agriculture workers. Shukla et al. considered the nasal and oral microbiomes of dairy farm workers and found increased diversity in dairy workers compared to urban controls and found dairy workers had less *staphylococci* in their nares [7]. Stanaway et al. assessed the oral (buccal swab) microbiome of agriculture workers exposed to pesticides and found organophosphate pesticide exposure significantly reduces microbial diversity that persists for extended periods of time [8].

The aim of this study was to assess the microbial composition of the anterior nares and oropharynx of livestock workers compared to those without livestock contact using culture-independent techniques. To our knowledge, our study is the first to assess the microbiomes of the anterior nares and oropharynx of healthy livestock workers from diverse agricultural backgrounds.

## Methods

### Ethics statement

All study protocols were approved by the University of Iowa Institutional Review Board (#201410803) prior to enrollment. Participants were enrolled into a cross-sectional study between April 2015 and March 2016 in Eastern Iowa. Eligibility criteria were: 18 years of age, English speaking, have not taken antibiotics or inhaled corticosteroids in the prior three months, not had the nasal influenza vaccine in the last month, no active infections of the upper respiratory tract, not hospitalized for greater than 24 hours in the last three months, and did not have HIV/AIDS. Study participants were informed about the risks of participation in the study. All participants provided written consent.

### Study population and enrollment

Participants were enrolled in one of four ways. First, through a pre-existing cohort consisting of 95 families (177 participants over 18 years of age) [9]. One individual from each family was contacted by letter and then by phone call to schedule enrollment. If the original contact person for each family was either not interested or ineligible for participation, a letter was sent to the other members of the family unit until all eligible adults in the cohort were contacted. Only one individual from each family unit was eligible for participation. Participants enrolled from the pre-existing cohort were both livestock workers and non-livestock workers.

Livestock workers were also enrolled through the Iowa Department of Natural Resources (DNR) Animal Feeding Operations (AFO) database [10], Iowa county fairs, and snowball sampling. Operations were chosen from eastern Iowa counties in the DNR AFO database and mailed an invitation letter. For the county fairs, a researcher passed out information on the study to livestock workers attending the fair. Participants could either take an information packet and contact the study team at a later date or could answer several eligibility questions and schedule an enrollment date while at the fair. Lastly, snowball sampling was used to recruit participants. Already enrolled livestock workers were asked to reach out to other livestock workers they knew (who did not live in their household and did not work on the same operation). Interested potential participants then called the study team to set up enrollment.

### Sample collection and processing

Enrollment occurred in the participant’s home. After consenting, participants filled out questionnaires assessing demographic characteristics, medical history, and animal contact (S1 File). We also requested participants not eat, drink, or brush their teeth within one hour of sample collection. Following the questionnaires, each participant provided swabs from their anterior nares and oropharynx. All samples were collected by a trained researcher and transported to the University of Iowa Center for Emerging Infectious Diseases (CEID) for processing. Samples were collected on sterile, dry, nylon flocked swabs (Copan Diagnostics, Murrieta, CA).

Bacterial DNA was isolated using the Qiagen PowerSoil DNA isolation kit (Qiagen Inc, Germantown, MD, USA) adapted for swab use by removing the swab head and placing it in the tube during bead beating. Negative controls (kit reagents only) were used for every batch of extractions. Samples were sent for sequencing (including library preparation) to the University of Minnesota Genomics Center. 16s rRNA sequencing of the v1-v3 region was carried out on the Illumina MiSeq using 2x300 nt reads. Briefly, DNA was normalized to 5ng/μL for amplicon polymerase chain reaction (PCR) followed by a PCR clean-up step using AMPure XP beads to prepare for indexing. Index PCR was then done to attach the dual indices and sequencing adapters using the Nextera XT Index kit followed by another PCR clean-up step and library validation. Fluorometry was used for library quantification followed by normalization and pooling. The library was diluted to 4 nM and 5 μl of diluted DNA was used for pooling. The library was then denatured (using NaOH and heat) and diluted to prepare for sequencing on the MiSeq using the v3 chemistry. Additional information on primers, PCR cycling conditions, and negative controls can be found in S2 File.

### Statistical analysis

The chi-square test and t-test were used to determine differences in participant characteristics between those with and without livestock exposure. The Fisher’s exact test was used when cell sizes were less than 5.

Sequences were assessed for quality using FastQC (Babraham Institute, Cambridge, UK) with poor quality reads filtered out (poor quality sequencing reads are defined as sequences with low base quality scores, short reads less than 200bp, reads with uncalled nucleotide bases, or any reads that could not assemble into paired reads). Reads were assembled using FLASh [11]. USEARCH version 8.1.1861[12] and Python version 2.7.12 were used for chimera removal, operational taxonomic unit (OTU) binning, and taxonomy assignment at the genus level following the UPARSE pipeline [12]. The Ribosomal Database Project (RDP) classifier was used as the reference database [13]. OTUs were grouped together based on 97% similarity. Any species level classification was done using BLAST+2.4.0 and the blastn function [14]. Human-associated OTUs were also removed from the dataset using BLAST+2.4.0 and the blastn function [14]. Further information on data processing can be found in S2 File. R version 3.3.1 was used for all statistical analyses and plot generation using the following packages: phyloseq [15], vegan [16], DESeq2 [17], and ampvis [18]. Alpha diversity was assessed using the Inverse Simpson diversity index [19] and beta diversity was assessed using the Bray-Curtis dissimilarity measure [20]. Principal coordinates analysis (PCoA) was used to visualize beta diversity. PERMANOVA [21], through the vegan package, was used to assess diversity differences between groups. The DESeq2 and ampvis packages were used to assess microbiota differences between groups. The DESeq2 package is only able to perform comparisons between two groups, as such animal contact was collapsed to swine versus all others when considering differentially abundant OTUs. To assess age and gender as confounders, we created subset datasets for those 55 and under and over 55 as well as males and females and compared the influence on livestock contact within each gender. Fifty-five was chosen as the age category cut off as roughly half of the participants were over 55 and half under. Additionally, 55 was in the middle of the mean ages for livestock workers and those with no livestock contact. The Benjamini-Hochberg correction for the false discovery rate was applied to all DESeq2 comparisons and adjusted p-values are reported. Results were considered significant if the *P* was less than 0.05 for all comparisons [22] with the exception of the DESeq2 analysis in where less than 0.01 was considered significant.

### Data access

The OTU table (DOI: 10.6084/m9.figshare.7538789), metadata (DOI: 10.6084/m9.figshare.7538792), and taxonomy classification (DOI: 10.6084/m9.figshare.7538780) files can be downloaded from figshare (https://figshare.com) and are part of the Livestock Worker Microbiome Project.

## Results

### Participant demographics

**Fig 1:**
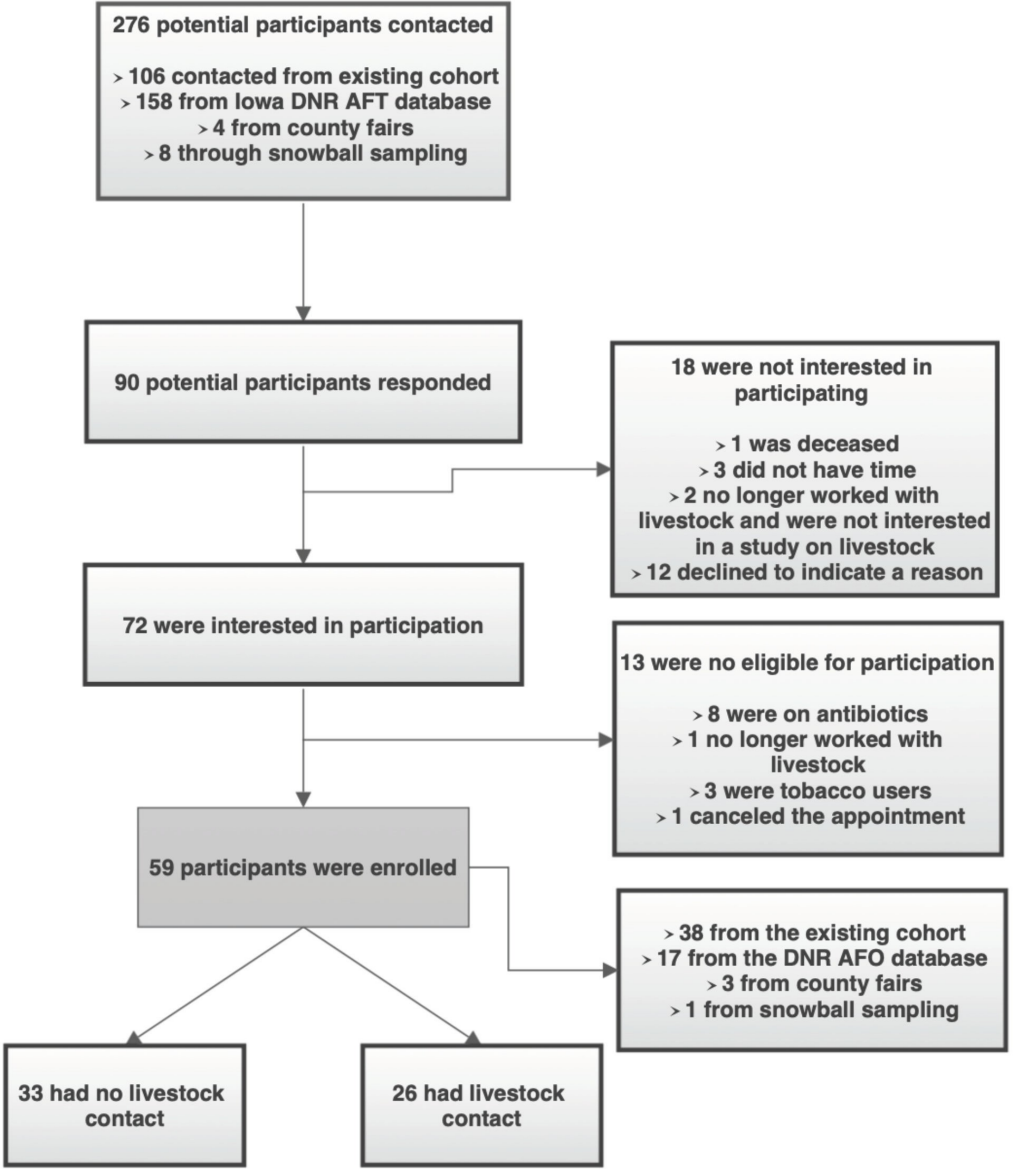
Flow diagram of participant enrollment.

Fifty-nine participants (26 livestock workers and 33 non-livestock workers) were enrolled (Fig 1). The average age of participants was 54.6 years (range: 28–85 years) and 41 (69.5%) were male. Livestock workers were significantly older than non-livestock workers (59.1 and 51.1 years respectively, *P* = 0.027) and were predominantly male (92.3%) while males only made up 51.5% of the non-livestock workers (*P* = 0.0007) (Table 1). Those without livestock contact were more likely to brush their teeth daily (*P* <0.001), use liquid hand soaps (*P* <0.001), and more likely to use a gym (*P =* 0.011) compared to those with livestock contact (Table 2). There were no other significant differences between those with and without livestock contact.

**Table 1:** Participant demographics.

	Livestock Contact (n = 26)	No livestock Exposure (n = 33)	p-value	Full Cohort (n = 59)
**Age (years)**	59.1	51.1	0.027	54.6
**BMI**	27.4	28.3	0.53	27.9
**Sex**				
**Male**	24 (92.3%)	17 (51.5%)		41 (69.5%)
**Female**	2 (7.7%)	16 (48.5%)	0.0007	18 (30.5%)
Race*				
**Caucasian**	25 (96.2%)	33 (100.0%)		58 (98.3%)
**Other**	1 (3.8%)	4 (12.0%)	0.394	5 (8.5%)
**Income (net)**				
**<$20,000**	0 (0.0%)	1 (3.0%)		1 (1.7%)
**$20,000-$39,999**	3 (11.5%)	5 (15.2%)		8 (13.6%)
**$40,000-$59,999**	3 (11.5%)	6 (18.2%)		9 (15.3%)
**$60,000-$79,999**	9 (34.6%)	12 (36.4%)		21 (35.6%)
**$80,000-$99,999**	6 (23.1%)	2 (6.1%)		8 (13.6%)
**>$100,000**	5 (19.2%)	7 (21.2%)	0.508	12 (20.3%)
**Highest level of education**				
**Less than high school**	0 (0.0%)	0 (0.0%)		0 (0.0%)
**High school graduate**	8 (31.7%)	3 (9.1%)		11 (18.6%)
**Some college**	3 (11.5%)	5 (15.2%)		8 (13.6%)
**College graduate**	12 (46.2%)	15 (45.5%)		27 (45.8%)
**Graduate level**	3 (11.5%)	9 (27.3%)		12 (20.3%)
**Professional level**	0 (0.0%)	1 (3.0%)	.174	1 (1.7%)
**House size**				
**<1500 sq. ft.**	5 (19.2%)	5 (15.2%)		10 (16.9%)
**>1500 sq. ft.**	19 (73.1%)	27 (81.8%)		46 (77.9%)
**Unknown**	2 (7.7%)	1 (3.0%)	0.693	3 (5.1%)
**Family Size**				
**1**	4 (15.4%)	2 (6.1%)		6 (10.2%)
**2**	13 (50.0%)	14 (42.4%)		27 (45.8%)
**3**	3 (11.5%)	5 (15.2%)		8 (13.6%)
**4**	2 (7.7%)	6 (18.2%)		8 (13.6%)
**≥5**	4 (15.4%)	6 (18.2%)	0.589	10 (16.9%)

**Table 2:** Health and hygiene characteristics of participants.

	Livestock Contact (n = 26)	No livestock Exposure (n = 33)	p-value	Full Cohort (n = 59*)
**Asthma**				
**Yes**	0 (0.0%)	3 (9.1%)		3 (5.1%)
**No**	25 (100.0%)	30 (90.0%)	0.25	55 (93.2%)
**COPD**				
**Yes**	1 (4.2%)	1 (3.1%)		2 (3.5%)
**No**	23 (95.8%)	32 (96.9%)	0.429	55 (96.5%)
**Heart Disease**				
**Yes**	2 (8.0%)	6 (18.2%)		8 (13.8%)
**No**	23 (.92%)	27 (81.8%)	0.445	50 (86.2%)
**Diabetes**				
**Yes**	1 (4.0%)	1 (3.0%)		2 (3.4%)
**No**	24 (96.0%)	32 (97.0%)	1.0	56 (96.6%)
**Cancer**				
**Yes**	1 (4.0%)	3 (10.0%)		5 (9.1%)
**No**	24 (96.0%)	26 (86.7%)		49 (89.1%)
**Don’t know**	0 (0.0%)	1 (3.3%)	0.158	1 (1.8%)
**Past Cigarette Use**				
**Yes**	2 (8.0%)	9 (27.3%)		11 (19.0%)
**No**	23 (92.0%)	24 (72.7%)	0.09	47 (81.0%)
**Past Cigar Use**				
**Yes**	0 (0.0%)	4 (12.1%)		4 (6.8%)
**No**	26 (100.0%)	29 (87.9%)	0.123	55 (93.2%)
**Past Chew User**				
**Yes**	2 (7.7%)	3 (9.1%)		5 (8.5%)
**No**	24 (92.3%)	30 (90.9%)	0.848	54 (91.5%)
**Dentures**				
**Yes**	0 (0.0%)	2 (6.3%)		2 (3.4%)
**No**	26 (100.0%)	30 (93.8%)	0.497	56 (96.6%)
**Tooth Brushing Frequency**				
**Every morning**	9 (34.6%)	26 (78.8%)		35 (59.3%)
**Every evening**	17 (65.4%)	15 (45.5%)		32 (54.2%)
**Most mornings**	2 (7.7%)	1 (3.0%)		3 (5.1%)
**Most evenings**	4 (15.4%)	4 (12.1%)		8 (13.6%)
**Some mornings**	6 (23.1%)	1 (3.0%)		7 (11.9%)
**Some evenings**	2 (7.7%)	1 (3.0%)		3 (5.1%)
**No mornings**	0 (0.0%)	0 (0.0%)		0 (0.0%)
**No evenings**	0 (0.0%)	0 (0.0%)	<0.001	0 (0.0%)
**Probiotic usage**				
**Yes**	3 (11.5%)	4 (12.1%)		7 (12.1%)
**No**	23 (88.5%)	29 (87.9%)	1.0	51 (87.9%)
**Type of Hand Soap**				
**Non-antibacterial, bar**	11 (42.3%)	12 (36.4%)		23 (40.0%)
**Non-antibacterial, liquid**	11 (42.3%)	17 (51.5%)		28 (47.5%)
**Antibacterial, bar**	11 (42.3%)	8 (24.2%)		19 (32.2%)
**Antibacterial, liquid**	11 (42.3%)	19 (57.6%)		30 (50.8%)
**Other**	1 (3.8%)	0 (0.0%)	0.001	1 (1.7%)
**Visited a Correctional Facility**				
**Yes**	1 (3.8%)	1 (3.1%)		2 (3.4%)
**No**	25 (96.2%)	32 (96.9%)	1.0	56 (96.6%)
**Outpatient surgery in last 3 months?**				
**Yes**	0 (0.0%)	1 (3.1%)		1 (1.7%)
**No**	26 (100.0%)	31 (96.9%)	1.0	57 (98.3%)
**Visited a hospital or long-term care facility?**				
**Yes**	14 (53.8%)	10 (31.3%)		24 (41.4%)
**No**	12 (46.2%)	22 (68.8%)	0.142	34 (58.6%)
**Work/ volunteer in a healthcare facility?**				
**Yes**	1 (3.8%)	7 (22.6%)		8 (14.0%)
**No**	25 (96.2%)	24 (77.4%)	0.59	49 (86.0%)

*Several participants opted not to answer several questions

Twenty-six participants had current exposure to livestock (Table 3). The majority of participants worked with swine (n = 18). Several participants currently worked with more than one type of animal with seven participants working with two animal types, two working with three animal types, and one participant working with six animal types (swine, poultry, cattle, sheep, goats, and horses). The most frequent combination of animal types was swine and cattle (n = 4).

**Table 3:** Livestock contact (n = 26).

Animal	N (%)	Ave. Number Animals (range)	Ave. Days per week (range)	Ave. Hours per day (range)
**Swine**	18 (69.2%)	3,024 (8–10,000)	6.0 d (2–7)	2.5 h (0.5–10)
**Cattle**	12 (46.2%)	191 (4–850)	6.4 d (1–7)	1.6 h (0.5–3)
**Poultry**	4 (15.4%)	1,644 (20–6,500)	7.0 d	1.0 h (0.25–2)
**Other**				
**Sheep**	4 (15.4%)	28 (10–50)	6.5 d (6–7)	1.4 h (0.25–3)
**Horses**	2 (7.7%)	6.5 (1–12)	6.5 d (6–7)	5.5 h (3–8)
**Goats**	1 (3.8%)	10	7.0 d	2.0 h

### Microbiota analysis

**Fig 2:**
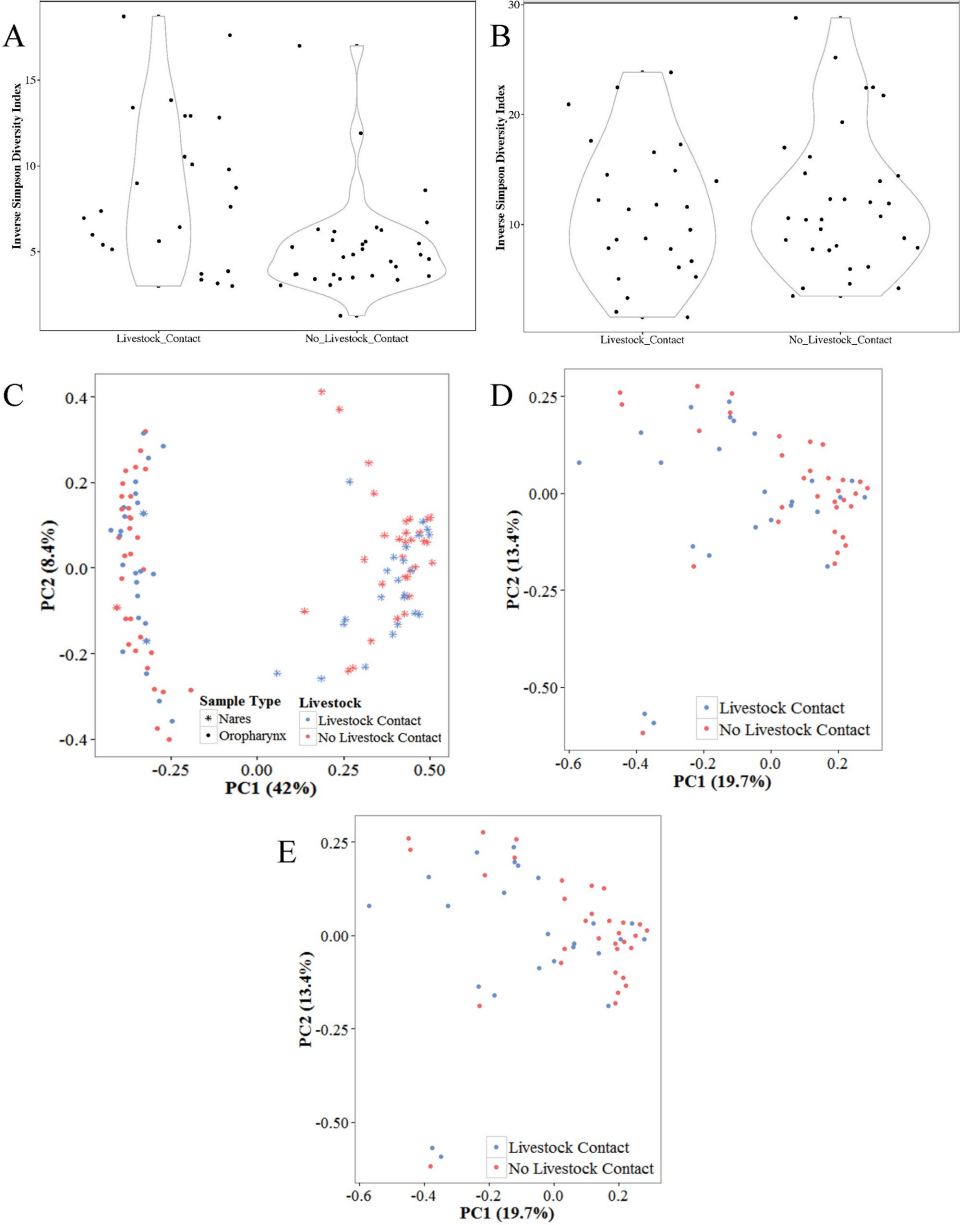
Diversity analysis by livestock contact. a) alpha diversity of nasal samples by livestock exposure (b) alpha diversity of oropharyngeal samples by livestock exposure. c) PCoA of the Bray-Curtis dissimilarity matrix all samples by livestock exposure. d) PCoA of the nasal samples. e) PCoA of the oropharyngeal samples. PC1 and PC2 = principle coordinates 1 and 2 respectively.

The Inverse Simpson diversity index (Fig 2) was greater for those with livestock contact compared to those without livestock contact in the nasal samples (p > 0.001)(Fig 2A); however, there was no difference in the oropharyngeal samples (p = 0.542) (Fig 2B). The ordination plot of the Bray-Curtis distances for all samples is shown in Fig 2C. The samples cluster by sample type (*P* = 0.001) and livestock exposure (*P* = 0.038, *P* for the interaction between sample type and livestock exposure p = 0.035). Because samples cluster by both livestock exposure and sample type, the nasal (Fig 2D) and oropharyngeal samples (Fig 2E) were assessed separately. A significant difference remained in the nasal samples (*P* = 0.002), but not the oropharyngeal samples (*P* = 0.559). There were no statistical differences in either alpha nor beta diversity based on any of the participant characteristics listed in Table 1.

There was no difference in alpha diversity by animal type (cattle, poultry, swine, more than one animal type) in either the nares (*P* = 0.762) or oropharynx (*P* = 0.941). When considering the beta diversity in the nares, there was a difference by animal types in the PCoA plot (*P* = 0.009); however, there were no differences in the oropharynx (*P* = 0.297).

Actinobacteria was present in every nasal sample and all but 3 oropharyngeal samples. Two of the three without Actinobacteria had no livestock contact and one did. Firmicutes were present in all but one nasal sample (this person had no livestock contact) and all of the oropharyngeal samples. Bacteroidetes was present in all but one oropharyngeal samples (the one sample without Bacteroidetes came from a livestock worker). In the nares, Bacteroidetes was only present in 16 of the samples, 13 of which came from those with livestock exposure. The barplot and boxplot of the most abundant OTUs can be found in the supplemental information (S1 Fig, S2 Fig).

**Fig 3:**
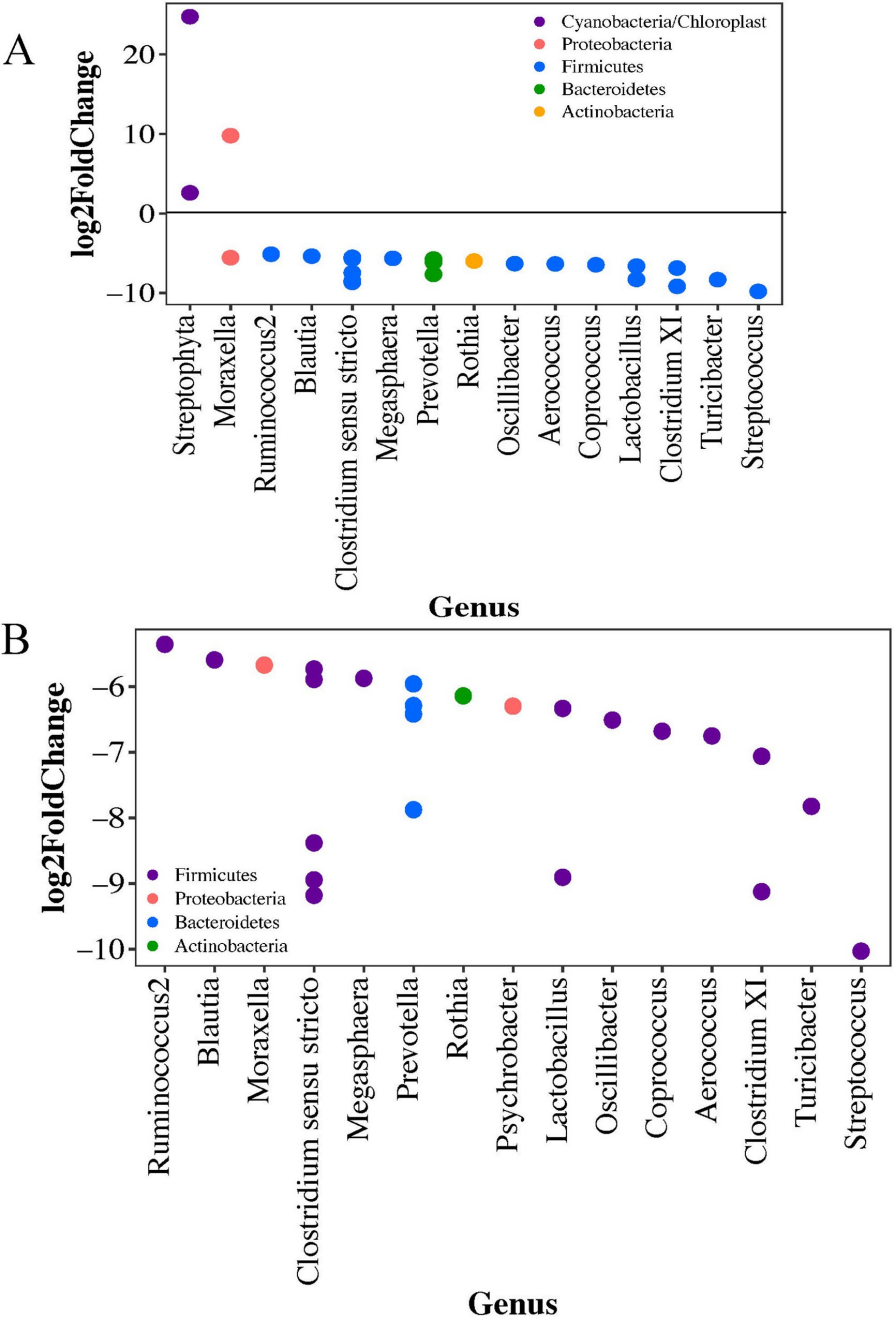
Log_2_-foldChange of the significantly differentially abundant OTUs in the nares by gender and livestock exposure. Points represent OTUs with phyla represented by color. Negative values represent OTUs significantly more abundant in livestock workers and positive values represent OTUs significantly more abundant in non-livestock workers. a) Differentially abundant OTUs in the nares of all participant by livestock and b) differentially abundant OTUs in the nares of male participants by livestock exposure. The Benjamini-Hochberg correction applied to all figures.

A total of 27 OTUs were differentially represented between the livestock workers and non-livestock workers, 24 of which were significantly more abundant in those with livestock contact in the nares (Fig 3A). When considering gender, only one OTU, belonging to *Simonsiella* genus, was significantly different (more abundant) in females nares without livestock exposure compared to those with livestock exposure. When looking at the male nares, 24 OTUs were significantly different between males with and without livestock exposure with all OTUs being more abundant in those with livestock contact (Fig 3B). Additional information including the base mean and Log_2_-fold change values can be found in S3 File.

**Fig 4:**
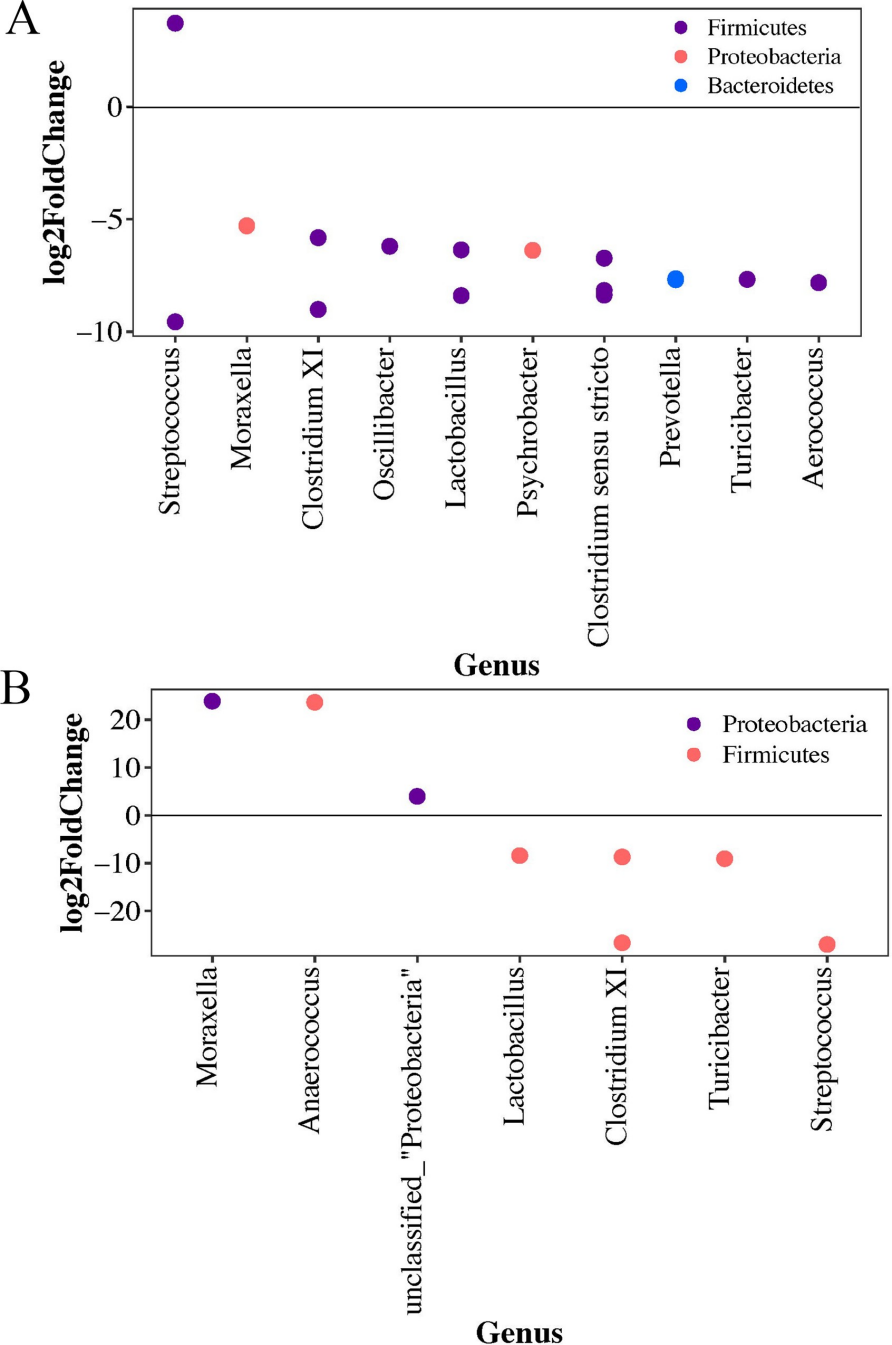
Log_2_-foldChange of the significantly differentially abundant OTUs by age category and livestock exposurePoints represent OTUs with phyla represented by color. Negative values represent OTUs significantly more abundant in livestock workers and positive values represent OTUs significantly more abundant in non-livestock workers. a) Differentially abundant OTUs in the nares of those over 55 and b) Differentially abundant OTUs in the nares of those 55 and under. The Benjamini-Hochberg correction applied to all figures.

When stratifying the nasal microbiome by age alone there were no differentially abundant OTUs. When further stratifying by age and livestock exposure, 16 OTUs were differentially abundant in the 55 and under and 8 OTUs in the over 55 groups. *Moraxella*, *Aerococcus*, *Lactobacillus*, *Clostridium XI*, *Turicibacter*, and *Strepotococus* remained differentially abundant across all comparisons. The only two OTUs identified by the age category analysis not observed in the gender analyses were *Psychrobacter* (more abundant in those over 55 with livestock contact) and an unclassified Proteobacteria (more abundant in those under 55 with no livestock contact). Several OTUs identified in the gender analysis were not identified as differentially abundant when stratifying by age: *Blautia*, *Streptophyta*, *Rumminococcus2*, *Megasphaera*, *Rothia*, and *Coprococcus* (Fig 4A, Fig 4B).

Unlike the nasal microbiome, there was a great deal of similarity between those with and without livestock contact in the oropharynx. There were no OTUs statistically more or less abundant between the livestock workers and those without livestock contact. When stratifying by gender, no OTUs were significantly different in the male oropharynx; however, one OTU belonging to *Treponema* was more significantly abundant in females with no livestock contact (Log_2_-fold change: 20.49, adjusted *p-*value: <0.001). When stratifying by age category and livestock exposure, there were no OTUs significantly differentially abundant in those under 55 by livestock exposure. In those over 55, individuals with livestock exposure were significantly more likely to carry *SR1 genera incertae sedis* in their oropharynx (Log_2_-fold change: -23.17, adjusted *p-*value: <0.001). The *Streptococcus* genus was the most prevalent genus observed in the oropharynx followed by *Prevotella* and *Haemophilus* genera.

**Fig 5:**
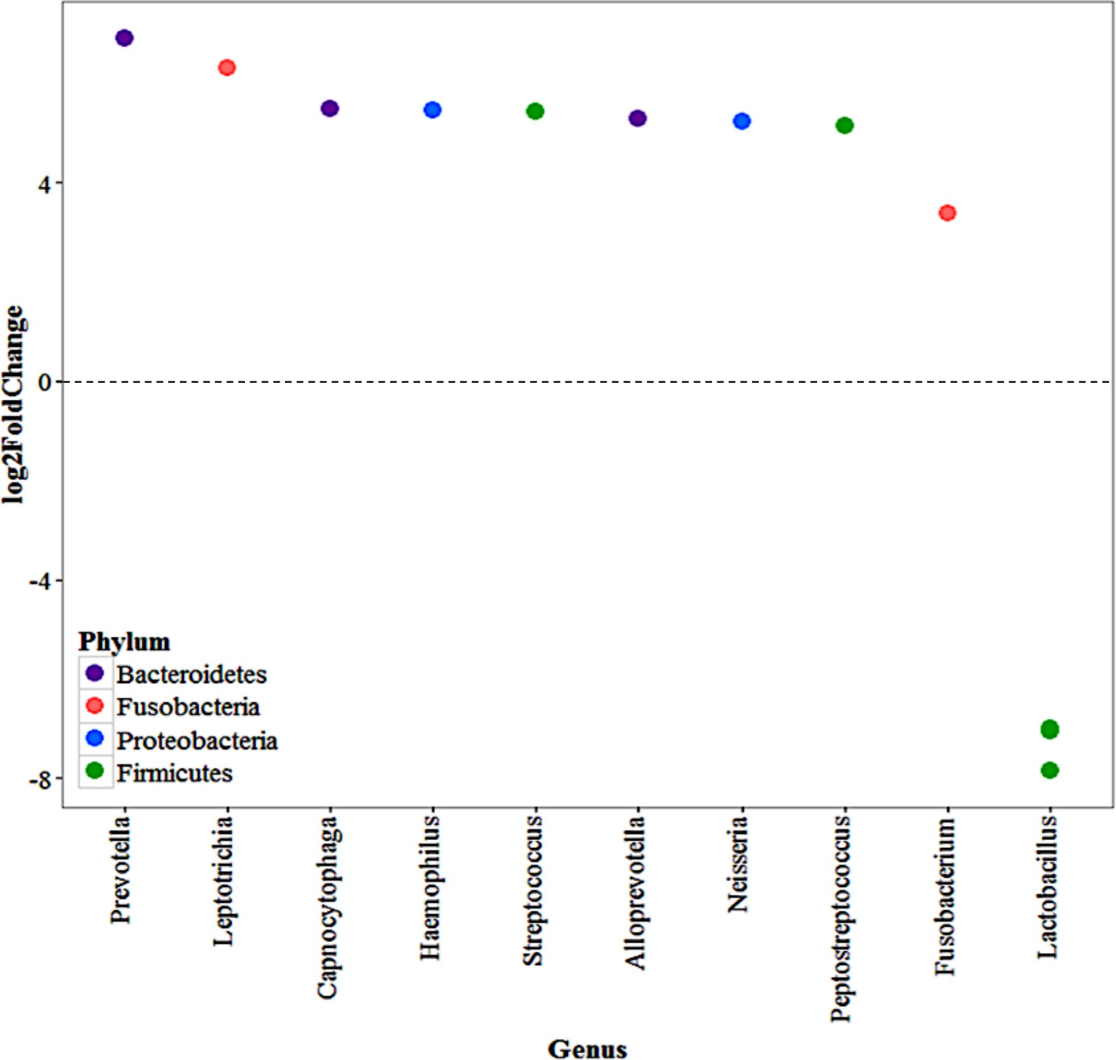
Log2-foldChange of the significantly differentially abundant OTUs in the oropharynx by animal contact (swine vs. all others) among those with livestock contact. Points represent OTUs with phyla represented by color. Negative values represent OTUs significantly more abundant in swine workers and positive values represent OTUs significantly more abundant in all other livestock workers. The Benjamini-Hochberg correction was applied.

When stratifying by animal type in the nares, *Corynebacterium* and *Staphylococcus* were the most abundant genera with members of the Firmicutes phylum being the most abundant (S3A Fig). When comparing swine workers to those with any other animal contact, one OTU was significantly more abundant in the swine workers, *Clostridium sensu stricto* (Log_2_-fold change: 8.58, *P* < 0.001). In the oropharynx there were nine OTUs significantly more abundant in the swine workers compared to those with all other animal types and three *Lactobacillus* OTUs with increased abundance in those with no swine contact (Fig 5). The boxplot of the top OTUs in the oropharynx can be found in S3B Fig.

A description of the negative controls can be found in S2 File. The negative controls had a much lower alpha diversity compared to the nasal and oropharyngeal samples andclustered separately from the nasal and oropharyngeal samples (*P* < 0.001). A total of 136 OTUs were identified in the negative controls.

## Discussion

Very little is known about the healthy livestock worker nasal and oropharyngeal microbiomes. The majority of studies assessing the microbial communities related to livestock work have either been done in animals [23, 24] or have studied the aerosolization of microorganisms in and around livestock facilities [5, 6, 25]. We are only aware of two studies to date considering the nasal and oral microbiomes of agriculture workers [7, 8]. Here we have described the nasal and oropharyngeal microbiomes of 26 livestock workers and 33 non-livestock workers in Iowa.

The population was comprised of primarily older (mean age of 54.6 years), Caucasian (98.3%) males (69.5%). Those with livestock contact were significantly older than those without livestock contact (59.1 years compared to 51.1 years) as well as more likely to be male (92.3% male compared to 51.5% male; respectively). This reflects the average farm worker in the United States where a majority of farm workers are males [26]. In the majority of Iowa counties, including Keokuk County, less than 10% of farm workers are female. Additionally, we observed no microbiota differences between males and females (data not shown). Furthermore, as of 2012, the average age of principal farmworkers was 58.3 years with more than half (61%) being between 35 and 64 years nationwide [26].

The importance of livestock contact on the human microbiome has been recognized in relation to respiratory diseases. It has been suggested that the farm effect has a protective effect on asthma. This is particularly true for children where early life exposure to microbes and microbial components educate the immune system by the upregulation of T-helper 1 cells and the downregulation of T-helper 2 cells reducing the risk of atopy [3]. Studies have shown having a parent in a farming occupation–particularly ones with livestock exposure–is significantly associated with lower rates of allergen disorders and allergy attacks and there is a dose response relationship with less atopy in children with parents who are full-time farmers [27, 28]. It is thought the high-diversity of microorganisms–likely inhaled–outcompete the harmful bacteria that may promote asthma [2]. In adults, asthma incidence is low (around 4%) as is atopy (14%) in farmers; however, unlike in children, asthma rates are higher among those who work with livestock, particularly swine and cattle [4]. Studies have also shown asthma to be more common in farmers without atopy than those with atopy and individuals with more than one type of animal exposure were at increased risk of non-atopic asthma [4].

Livestock workers had significantly more diverse nasal microbiomes compared to non-livestock workers, as observed by others [7], though this was not true for the oropharynx. One reason we may not have observed a difference in the oropharynx is potentially due to inhalation. Livestock workers are exposed to high levels of inhalable dust which contains microorganisms [5, 6]. The *Ruminococcaceae* family and *Lactobacillus* were both found to be significantly more abundant in the nares of those participants with livestock contact than those lacking this exposure and have been identified in inhalable dust [29]. *Moraxella–*a human commensal also known to cause respiratory tract infections [30]–is a bacterial air contaminant in livestock houses [31]. Others have found organisms belonging to the *Aerococcaceae* family, *Dietzia*, and *Prevotella* in air surrounding livestock [25]. OTUs belonging to all of these genera were significantly more abundant in the nares of those with livestock contact in our population leading to the conclusion these organisms are putatively associated with inhalation in the farming environment.

We identified several potential pathogens as more abundant in livestock workers’ nares and oropharynx in our population. Potential pathogens found in higher abundance in male livestock workers were *Prevotella* [32–34], *Streptococcus* [35–37], *Moraxella* [38, 39], *Rothia* [40], and *Oscillibacter* [41]. While the *Oscillbacter*, *Moraxella*, *Lactobacillus*, *Prevotella*, *Streptococcus*, and *Rothia* genera were observed in the negative control samples, they were not the same OTUs identified by the DESeq2 analysis. The only OTU identified in the DESeq2 analysis also identified in the negatives controls was OTU 17 (*Moraxella)* and was only found in 2 participants. While a number of OTUs were different between males with and without livestock exposure in the nares, only one OTU was differential when considering females. Again, when considering the oropharynx, females had one differential OTU. One reason for these differences in only 2 females reported any livestock exposure which is not a large enough sample size to make any accurate conclusions. When stratifying by age category and livestock, several of the OTUs identified by the livestock and gender by livestock stratifications were no longer present suggesting these OTUs may be more related to the participant’s age rather than their exposure to livestock.

Several *Prevotella* OTUs were identified as differentially abundant between males with and without livestock exposure and those over 55 with livestock exposure in the nares. *Prevotella* spp., particularly *P*. *ruminicola*, are difficult to culture microorganism prevalent in the gastrointestinal tracts of all livestock animals in addition to ruminants. It has been demonstrated *P*. *ruminocola* has the ability to transfer tetracycline resistance to other members of the Bacteroidetes phylum, particularly to other *Prevotella* species, in the host and horizontal transfer of the *tetQ* gene among *Prevotella* spp. is common in the human and ruminant intestines as well as the human oral cavity [42]. While it was not significantly enhanced in the livestock worker microbiome, *P*. *ruminocola* was present, as were many oral-associated *Prevotella* species. *Prevotella* spp. are frequent causes of odontogenic infections associated with gram-negative, anaerobic bacteria [43, 44]. These organisms are also known to cause infections of the respiratory system, head, and neck [44]. This is of interest as tetracycline is still commonly used in agriculture as well as a treatment for periodontal disease [45, 46] and *Prevotella* spp. were very common in the nares and oropharynx in our population and significantly more abundant in the oropharynx of swine workers.

As it is likely these organisms are being inhaled while working around livestock, it is possible their presence is transient contamination and not true colonization. While there is little research surrounding contamination vs. colonization, several studies have been done with regard to livestock worker colonization with *S*. *aureus* and have found many livestock workers drop *S*. *aureus* carriage within 24 hours [47]. On average it had been roughly 30 hours since swine workers had their last contact with swine, 24 hours since cattle workers had their last contact with cattle, and 1.5 hours since poultry workers had their last contact with poultry at the time of swabbing. It is possible some of the organisms observed in the nasal microbiome were due to contamination from recently being around their livestock, especially in those with poultry contact. As many of the swine and cattle workers were close to 24 hours since their last contact with animals, it is difficult to determine if the presence of these organisms is true colonization or temporary contamination without further longitudinal research.

We observed three participant behaviors to be significantly different between those with and without livestock contact: type of soap used, gym usage, and the frequency of tooth brushing. However, none of these behaviors were significantly associated with alterations in either the nasal or oropharyngeal microbiomes. The most surprising of these was that frequency of tooth brushing, which was less frequent in the livestock workers, but was not associated with any differences in oral microbiota. One explanation for this is frequency of tooth brushing may not be an adequate marker of oral hygiene. A better marker for oral hygiene may have been to assess the number of dental carries, gingivitis, gum disease, and/or halitosis. Future studies should assess oral hygiene using a standardized survey, such as the NHANES Oral Health Survey [48]; however, this study was not designed to specifically address this research question.

Our study is the first we are aware of to assess the microbiome of livestock workers using non-culture based methodologies and a great deal of additional research is needed. More research is needed to better understand the relation of the livestock worker respiratory microbiomes and diseases such as asthma. Longitudinal studies should be done to first characterize the livestock workers over time and at different stages of life. Animal-based studies are needed to more definitively assess the relationship between the core microbes of the livestock worker airways and their impact on COPD and other respiratory conditions.

## Supporting information

S1 File Study documents. This file contains the questionnaires developed for this study and filled out by participants.
(DOCX)

S2 File Additional sequencing methods and data. This file contains additional information and figures on the primers and PCR cycling conditions used, more in-depth descriptions of methodologies, and a description of the negative controls as well as what OTUs were present in the negative control samples.
(DOCX)

S3 File DESeq2 results. This file contains table with additional output from the DESeq2 results.
(DOCX)

S1 Fig Barplot of the most abundant phyla by livestock exposure across all samples. (TIFF)

S2 Fig Boxplot of the top 15 OTUs. (a) nares of those with and without livestock exposure and (b) oropharynx of those with and witout livestock exposure. Phylum and genus classification are shown. Percent abundances are log transformed.
(TIFF)

S3 Fig Boxplot of the top 15 OTUs in livestock workers by type of animal contact. a) nares b) oropharynx. Phylum and genus classification are shown. Percent abundances are log transformed.
(TIFF)
