# Supplementary figures and images for: The nasal and oropharyngeal microbiomes of healthy livestock workers
Source: PLoS One. 2019 Mar 12;14(3):e0212949. doi: 10.1371/journal.pone.0212949 (PMC6413945; doi:10.1371/journal.pone.0212949)

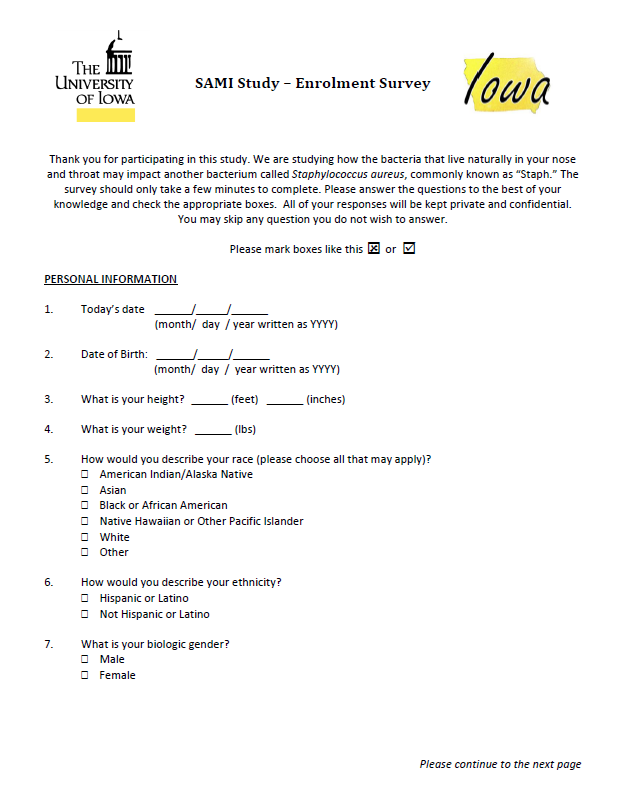


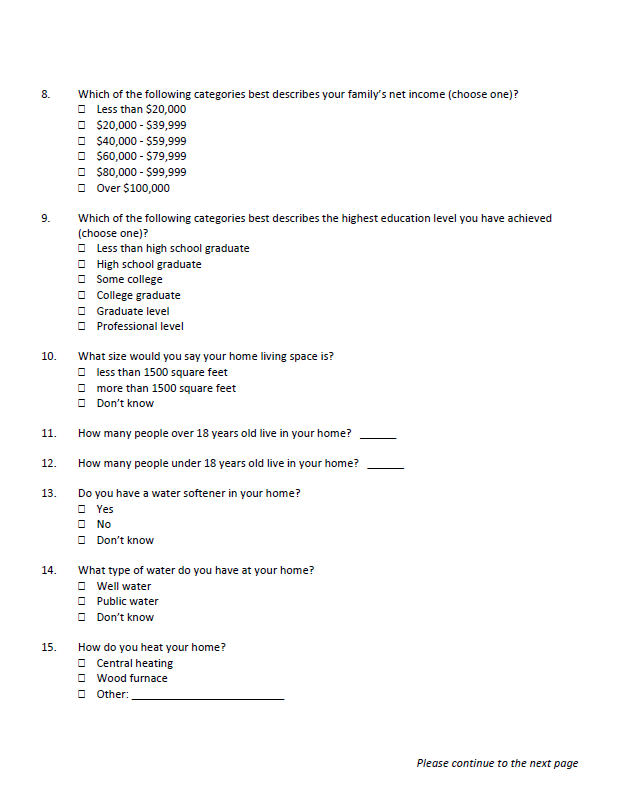


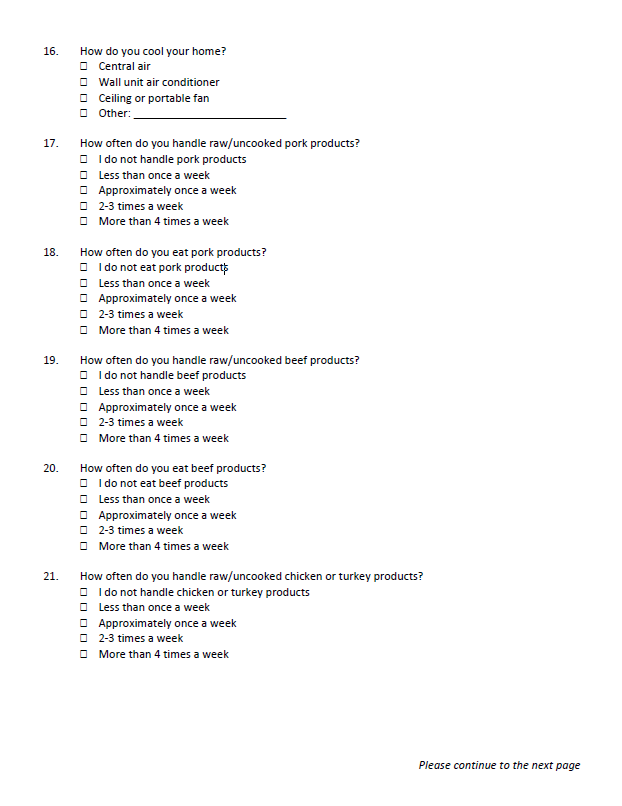


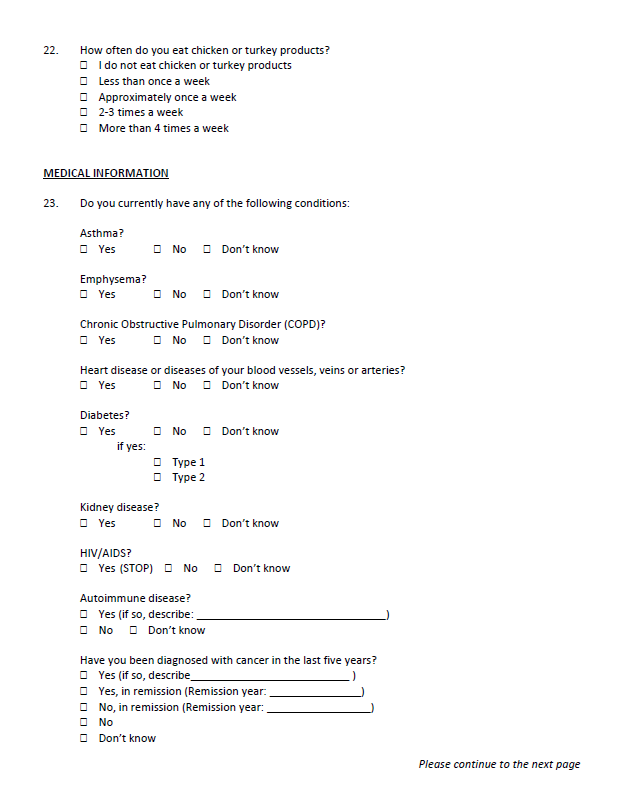


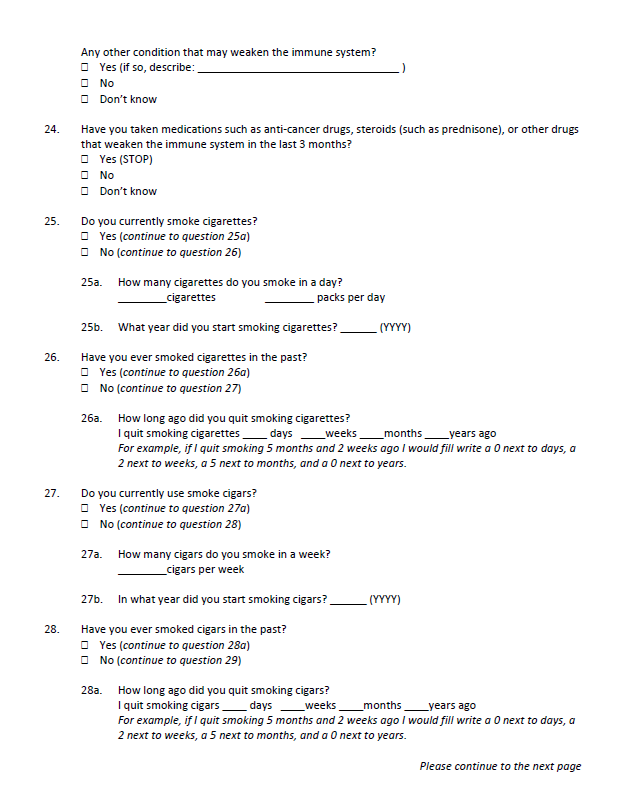


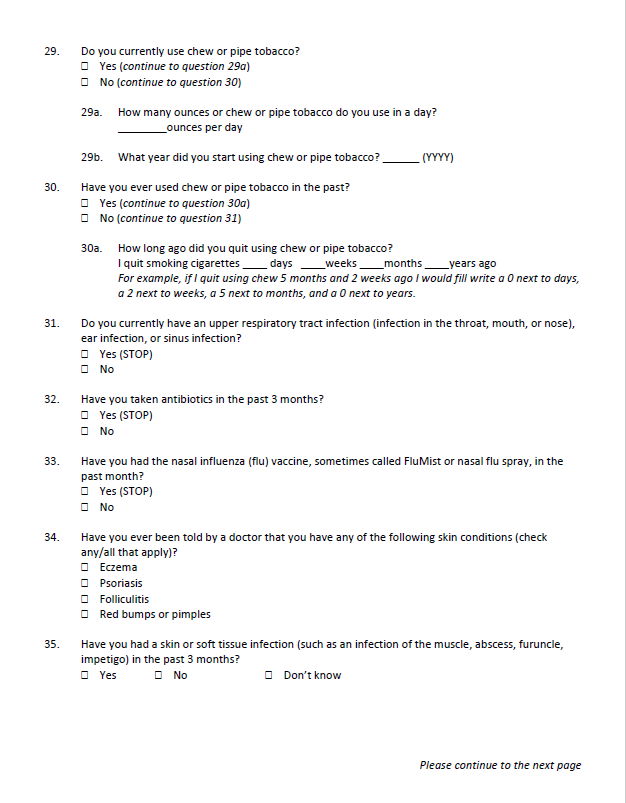


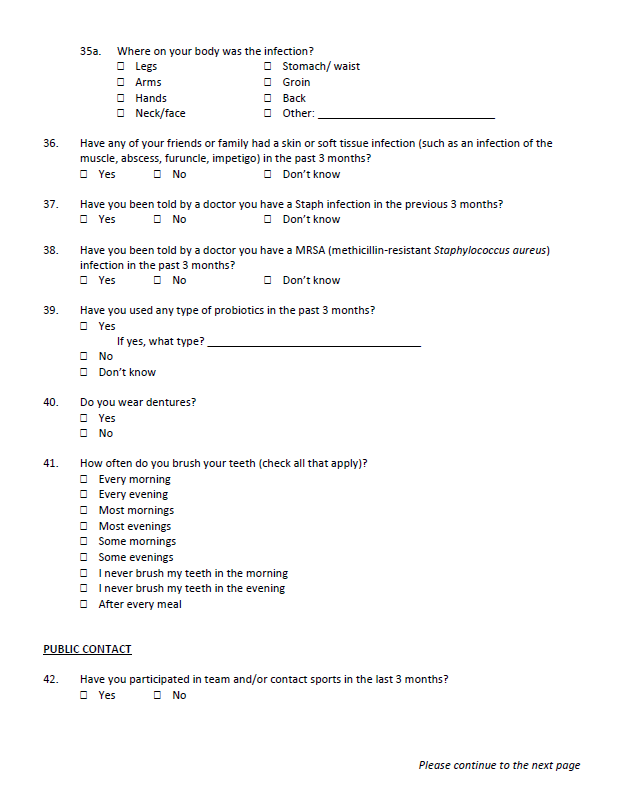


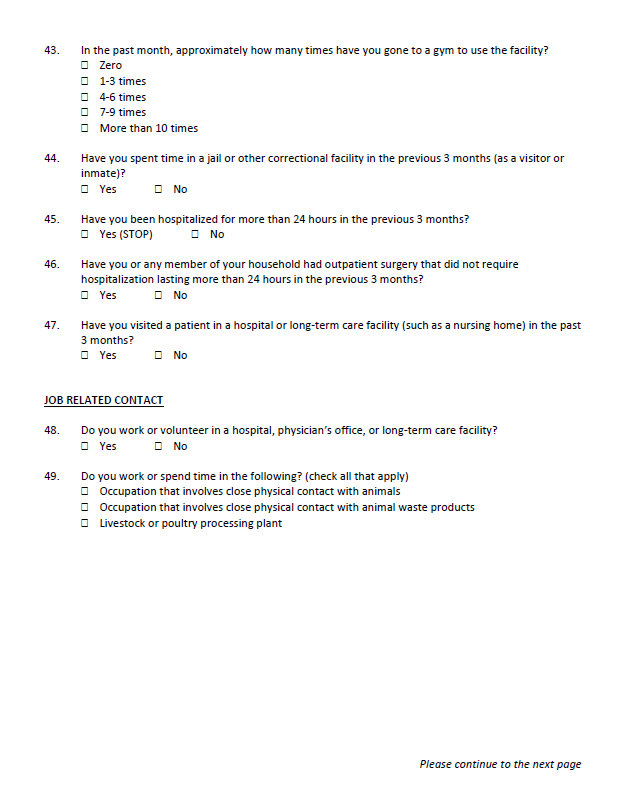


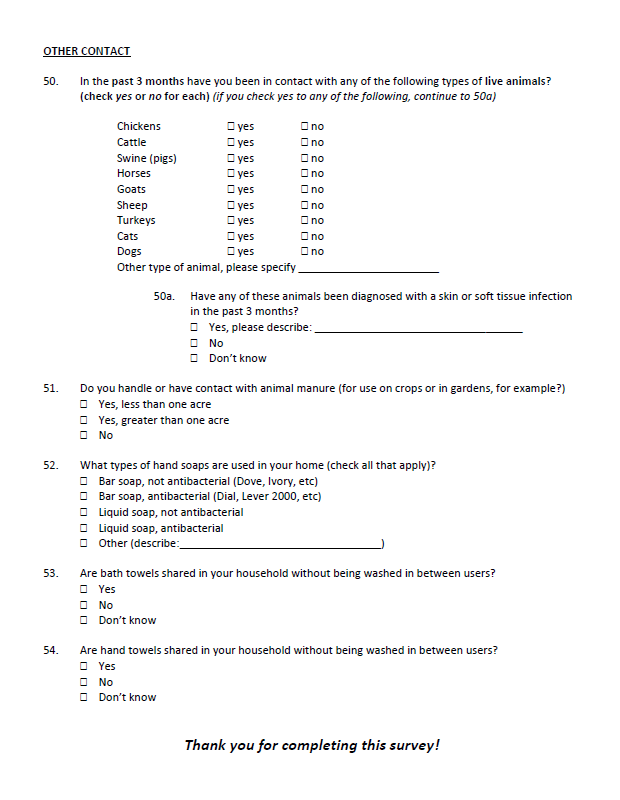


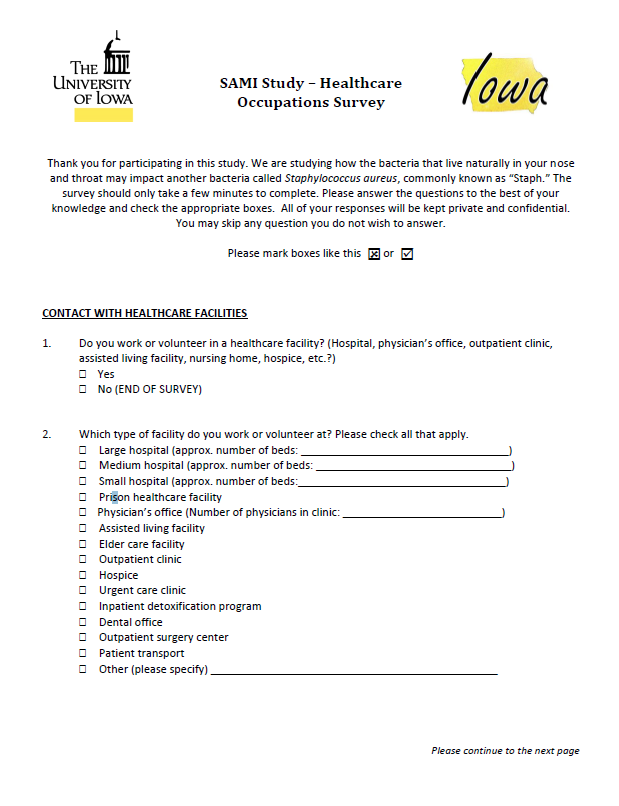


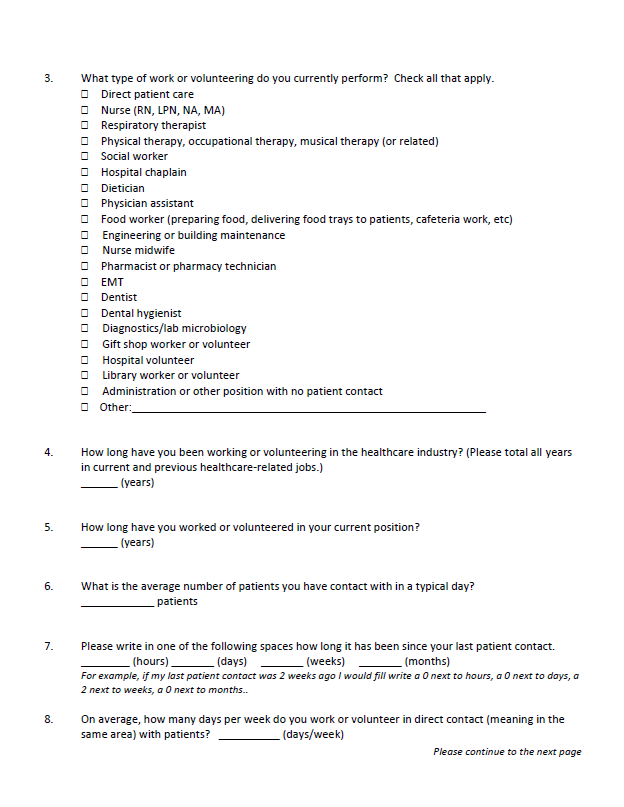


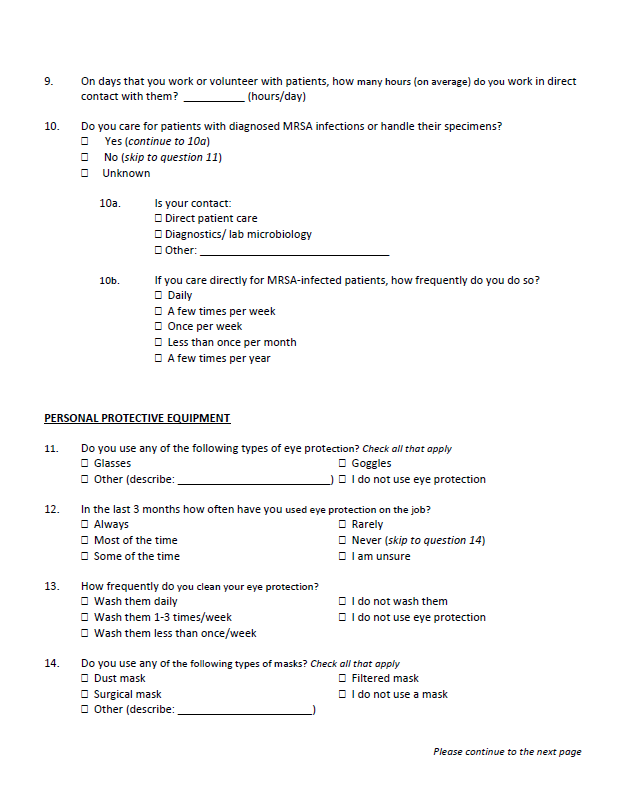


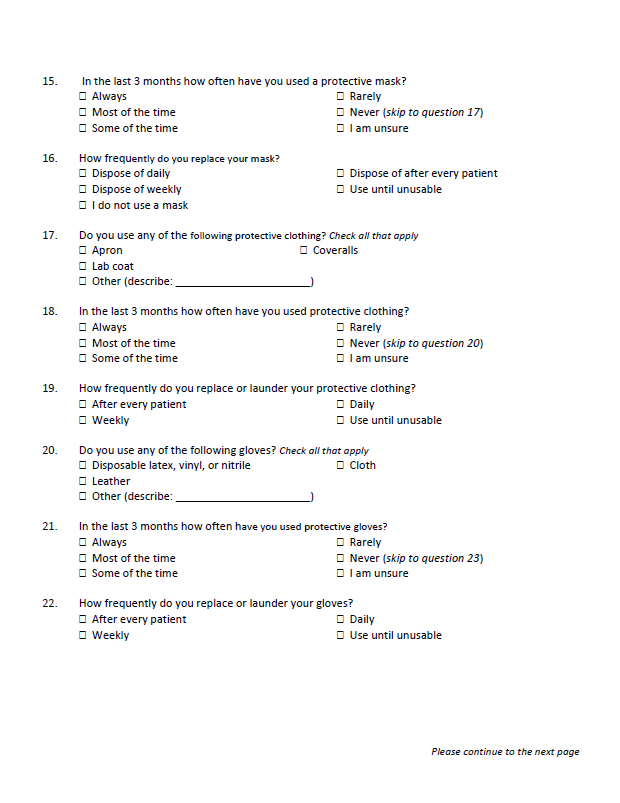


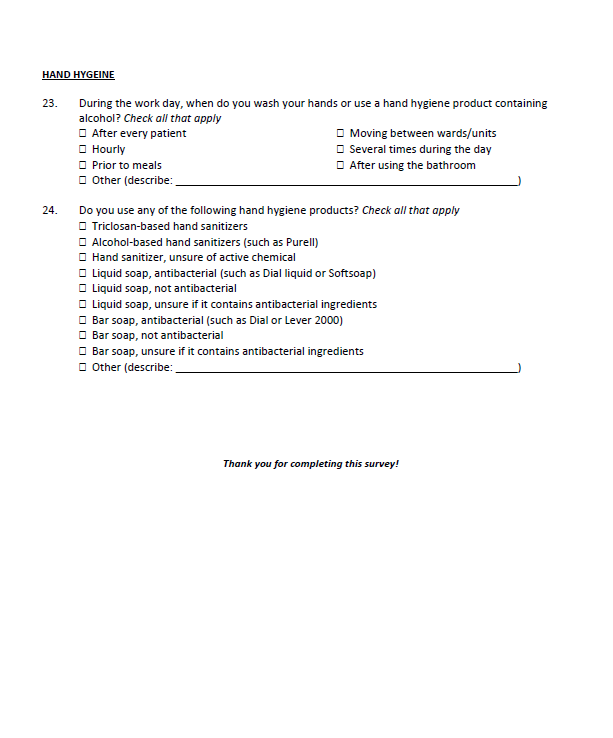


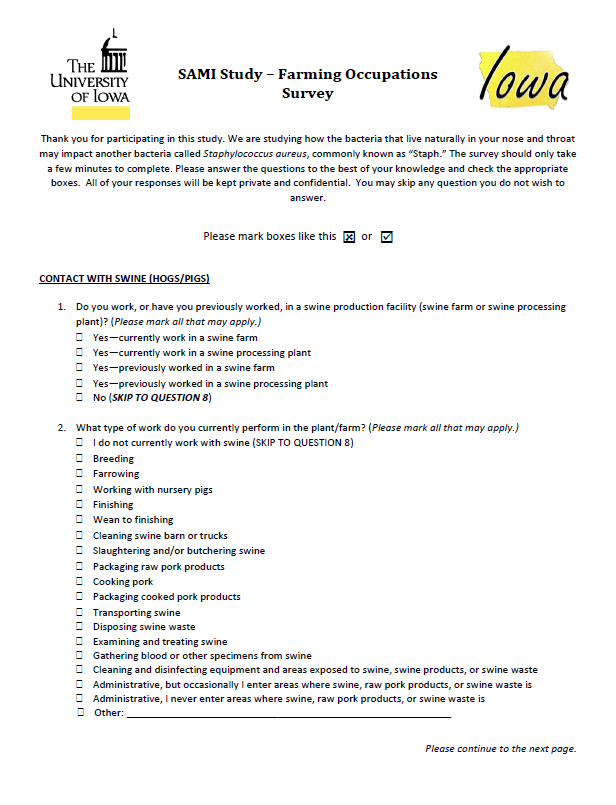


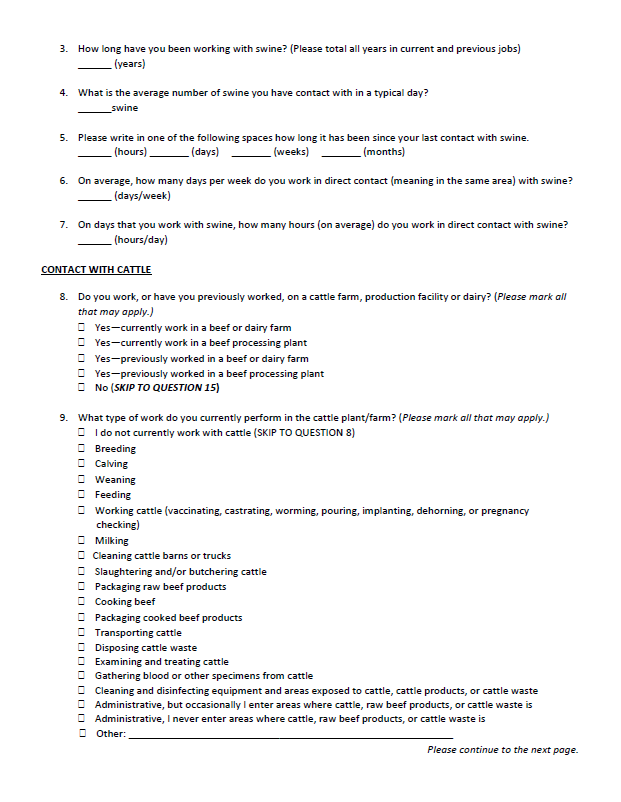


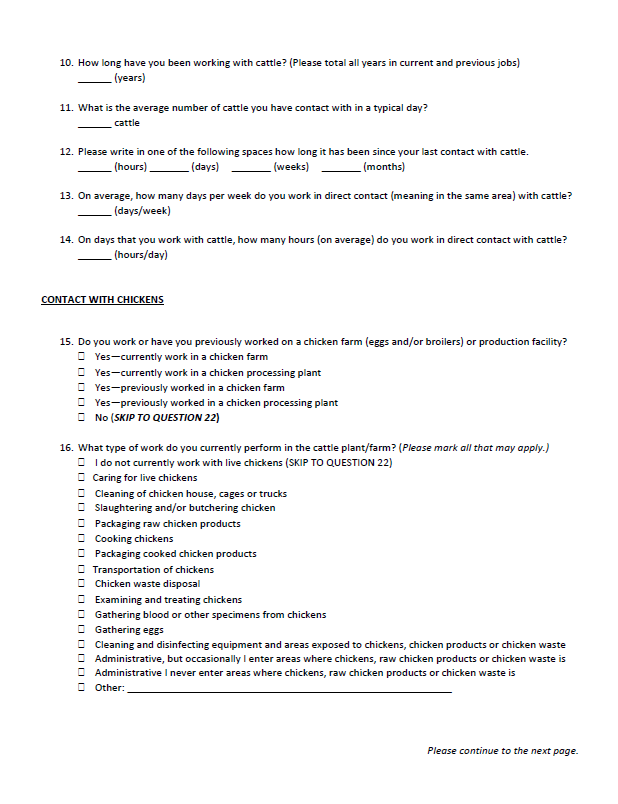


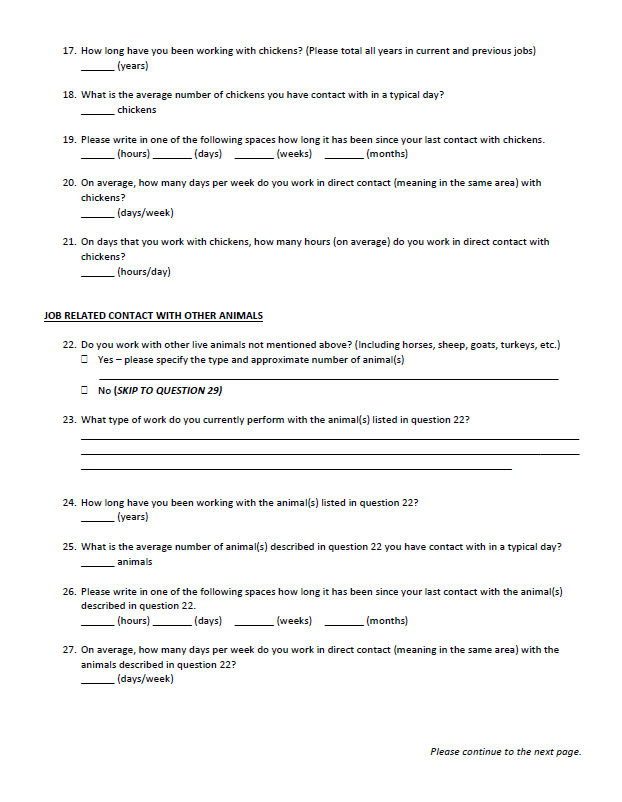


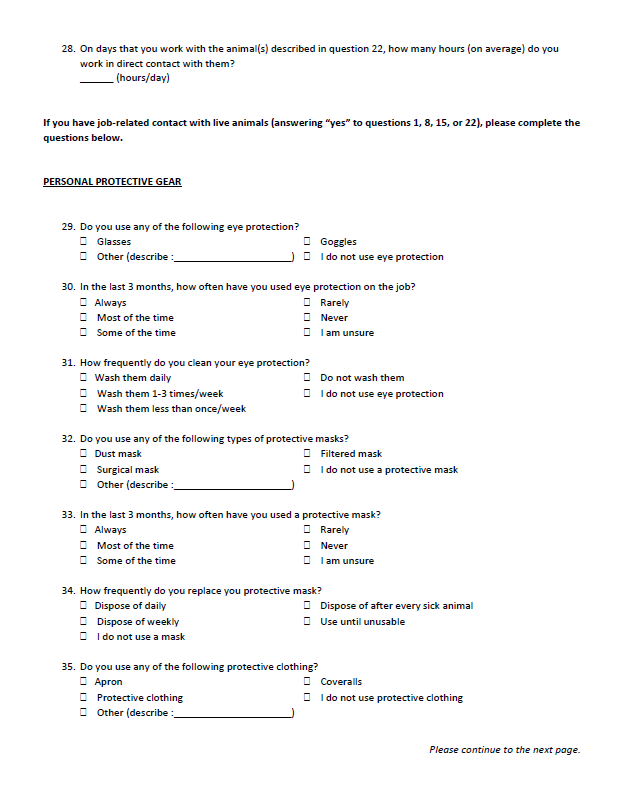


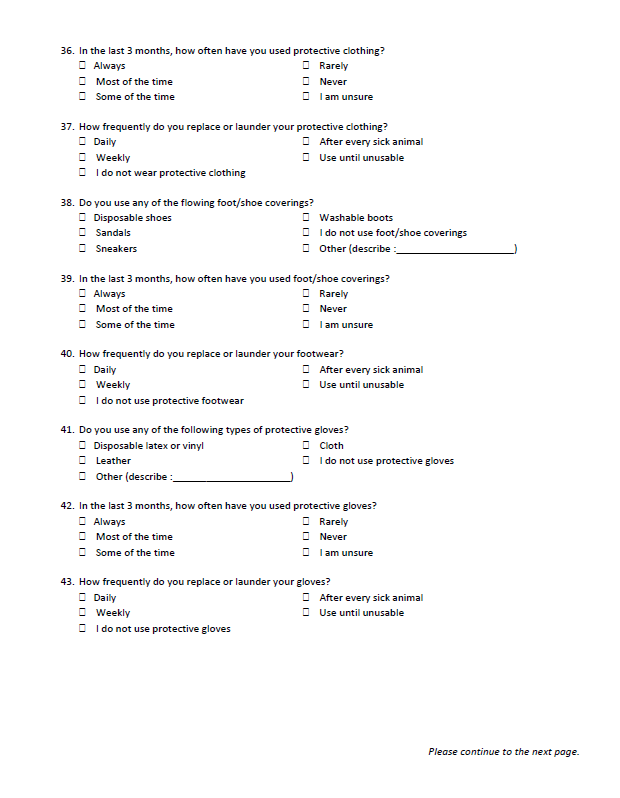


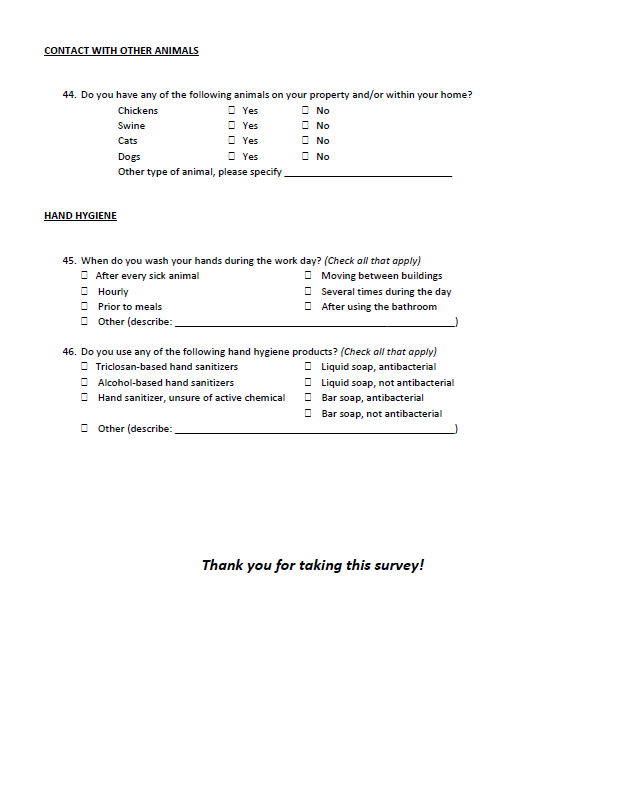

Supplement: S1 File — This file contains the questionnaires developed for this study and filled out by participants. (DOCX) [file pone.0212949.s001.docx]

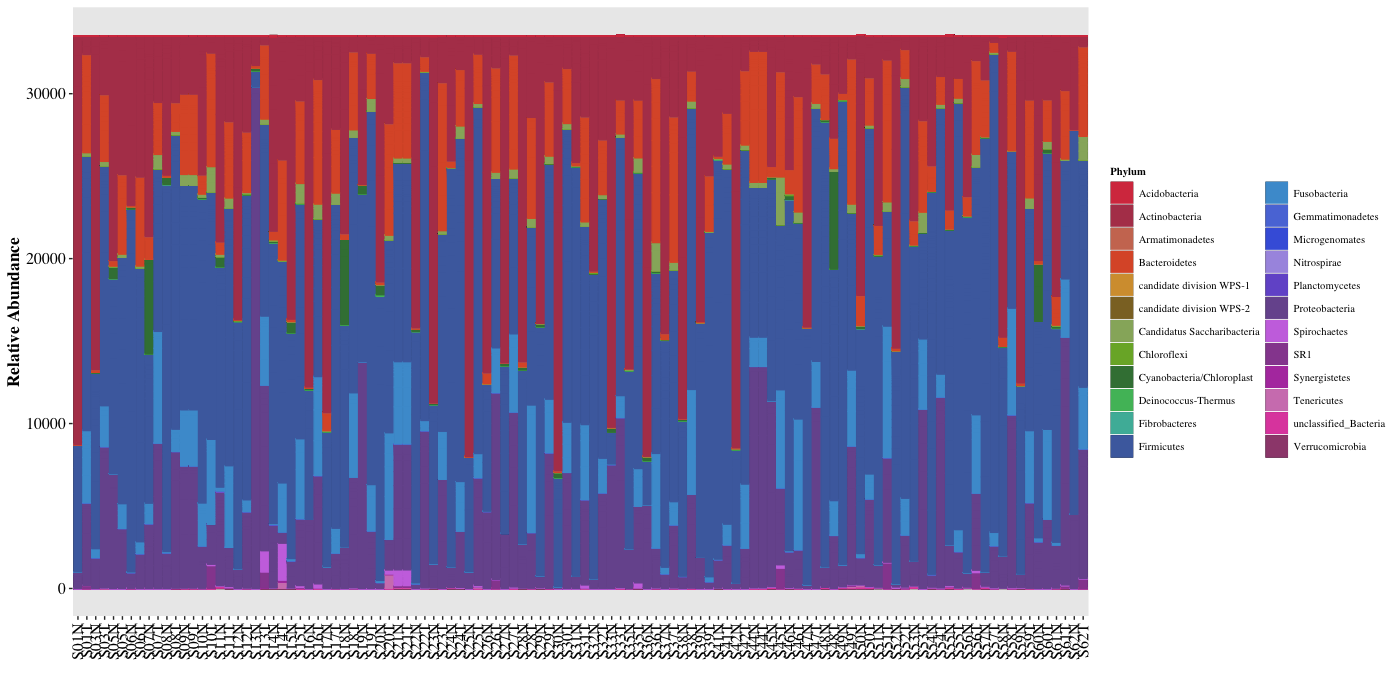

Supplement: S1 Fig — (TIFF) [file pone.0212949.s004.tiff]

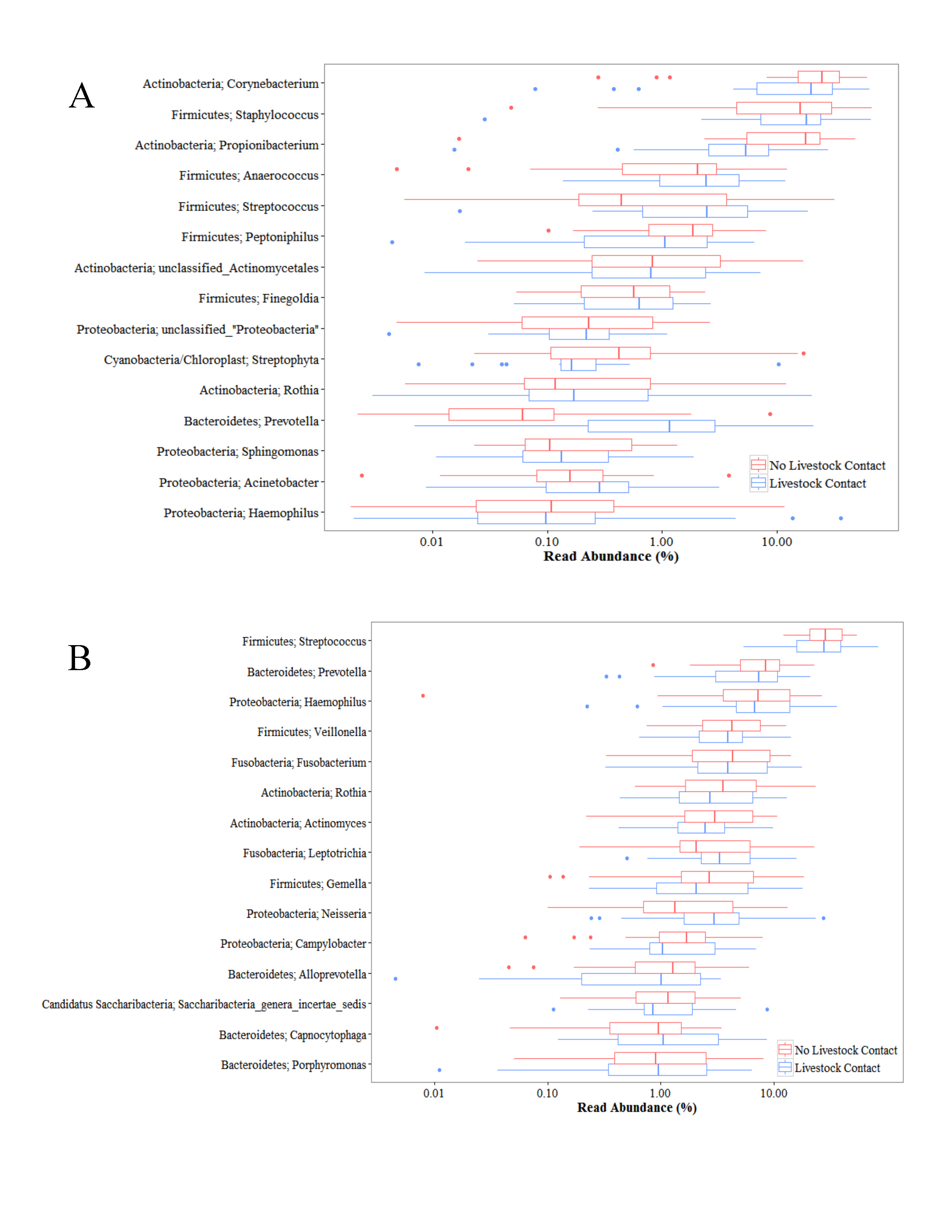

Supplement: S2 Fig — (a) nares of those with and without livestock exposure and (b) oropharynx of those with and witout livestock exposure. Phylum and genus classification are shown. Percent abundances are log transformed. (TIFF) [file pone.0212949.s005.tiff]

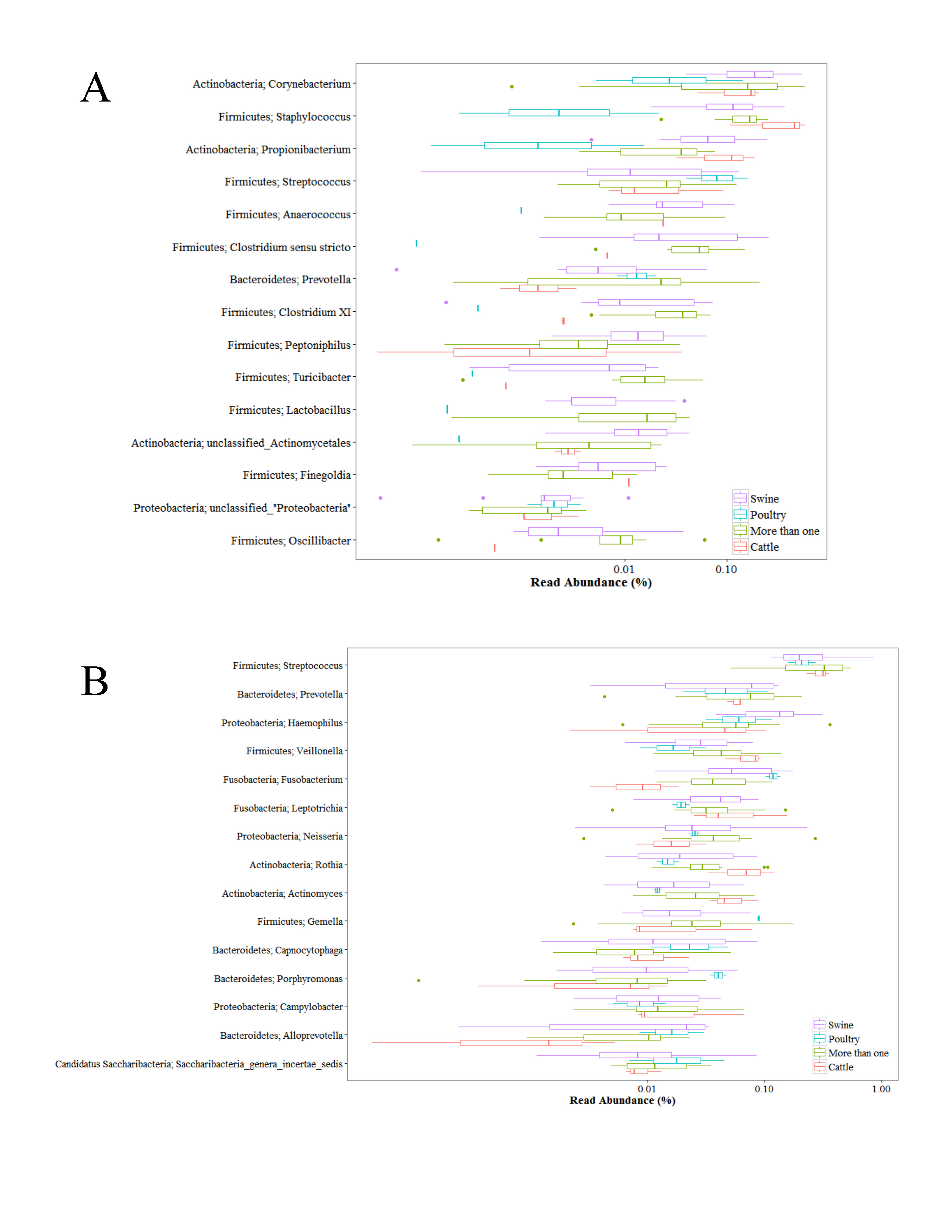

Supplement: S3 Fig — a) nares b) oropharynx. Phylum and genus classification are shown. Percent abundances are log transformed. (TIFF) [file pone.0212949.s006.tiff]
