# Supplementary material for: The nasal and oropharyngeal microbiomes of healthy livestock workers
Source: PLoS One. 2019 Mar 12;14(3):e0212949. doi: 10.1371/journal.pone.0212949 (PMC6413945; doi:10.1371/journal.pone.0212949)
Supplement: S2 File — This file contains additional information and figures on the primers and PCR cycling conditions used, more in-depth descriptions of methodologies, and a description of the negative controls as well as what OTUs were present in the negative control samples. (DOCX) [file pone.0212949.s002.docx]

Table S2-1: Primer sequences used for 16s rRNA sequencing

| Primer | Step | Sequence |
| --- | --- | --- |
| Meta V1 27F* | 16s rRNA amplification | TCGTCGGCAGCGTCAGATGTGTATAAGAGACAG**AGAGTTTGATCMTGGCTCAG** |
| Meta V3 534R* | 16s rRNA amplification | GTCTCGTGGGCTCGGAGATGTGTATAAGAGACAG**ATTACCGCGGCTGCTGG** |
| Forward index^§^ | Library coding | **AATGATACGGCGACCACCGA**GATCTACAC[i5]TCGTCGGCAGCGTC |
| Reverse index^§^ | Library coding | **CAAGCAGAAGACGGCATACGA**GAT[i7]GTCTCGTGGGCTCGG |
| Nextera adapter sequence | Read 1 post-run trimming | CTGTCTCTTATACACATCTCCGAGCCCACGAGACNNNNNNNNATCTCGTATGCCGTCTTCTGCTTG |
| Nextera adapter sequence | Read 2 post-run trimming | CTGTCTCTTATACACATCTGACGCTGCCGACGANNNNNNNNGTGTAGATCTCGGTGGTCGCCGTATCATT |

* 16S-specific portion of primer sequence is in bold.

^§^ [i5] and [i7] refer to the index sequence codes used by Illumina. The flow cell adapters are in bold.

## PCR Cycling Conditions

The following cycling conditions were used for the first PCR (amplification) using the Meta_V1_27F and Meta_V3_534R primer pair. Five minutes at 95°C followed by 15-25 cycles of: 98° for 20 seconds, 55°C for 15 seconds, and 72°C for 60 seconds; and holding at 4°C. After the first round of amplicon PCR, PCR products were diluted 1:100 and 5µl of 1:100 amplicon PCR product was used for the second round of PCR (PCR 2). Cycling conditions for PCR 2 were: 95°C for five minutes, 10 cycles of: 98° for 20 seconds, 55°C for 15 seconds, and 72°C for 60 seconds; and holding at 4°C.

**Sequencing clean-up and OTU binning**

Once sequencing is complete, raw data files was downloaded from a secure server (BaseSpace, Illumina). Once data is obtained from the University of Minnesota, data was assessed for quality and poor-quality data was filtered out (poor quality sequencing reads are defined as sequences with low base quality scores, short reads less than 200bp, reads with uncalled nucleotide bases, or any reads that could not assemble into paired reads). FastQC (Babraham Institute, Cambridge, UK) was used to asses sequence quality. Reads were assembled using FLASh with the following parameters: minimum overlap = 30, maximum overlap = 150, and mismatch = 0.1 (1). Adapters were removed from the merged file using Cutadapt (2). USEARCH version 8.1.1861 and Python version 2.7.12 were used for chimera removal, operational taxonomic unit (OTU) creation, and taxonomy assignment at the genus level following the UPARSE pipeline guidelines (3). The Ribosomal Database Project (RDP) classifier was used as the reference database (4). OTUs were grouped together based on 97% similarity. Species level classification was done for all *Staphylococcus*- associated OTUs as well as several of the differentially represented OTUs using BLAST+2.4.0 and the blastn function (5). Human-associated OTUs were also removed from the dataset using BLAST+2.4.0 and the blastn function (5).

**DESeq2 Log_2_ Fold Change Calculation**

The log_2_ fold changes and differential abundances were calculated using the following code in R with the DESeq2 and phyloseq packages.

newobject = phyloseq_to_deseq2(Phyloseq Object, ~ Variable)

gm_mean = function(x){exp(sum(log(x[x > 0]) / length(x)))}

geoMeans = apply(counts(newobject), 1, gm_mean)

newobject = estimateSizeFactors(newobject, geoMeans = geoMeans)

newobject = DESeq(newobject, fitType="local")

**Additional Run Data**

One thousand six hundred and eighty-two operational taxonomic units (OTUs) were identified in the dataset, of which were singletons. The majority of OTUs belonged to the Firmicutes phylum (n=574, 34.1%) followed by Proteobacteria (n=324, 19.3%), Bacteroidetes (n=296, 70, 17.6%), and Actinobacteria (n=293, 17.4%). Twenty-seven OTUs were unclassified bacteria (1.6%). The mean sequencing depth (number of reads) per sample was 30,310 with a range of 1,579 to 64,700 (Figure S2-1).

Negative controls were sequenced to assess if contamination was present in any of the laboratory reagents. Extraction controls (all reagents and kit components plus a sterile swab head), PCR controls (regents plus sterile, nuclease free water), and sequence run controls (regents plus sterile, nuclease free water) were used during each respective step. The alpha diversity of the samples by sample type (nasal, oropharyngeal, and negative control) is shown in Figure S2-2a. PCoA of the Bray-Curtis distances are shown in Figure S2-2b. Negative controls clustered separately from the nose and throat samples (*P* < 0.001). One hundred and thirty-six OTUs were identified in the negative controls (Figure S2-3). A barplot of the relative abundances of the negative controls can be found in Figure S2-4.


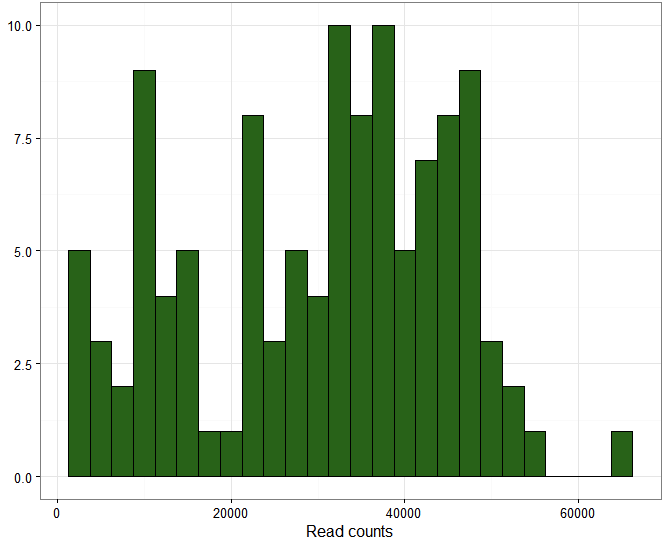


Figure S2-1: Barplot of sequencing depth for all samples.


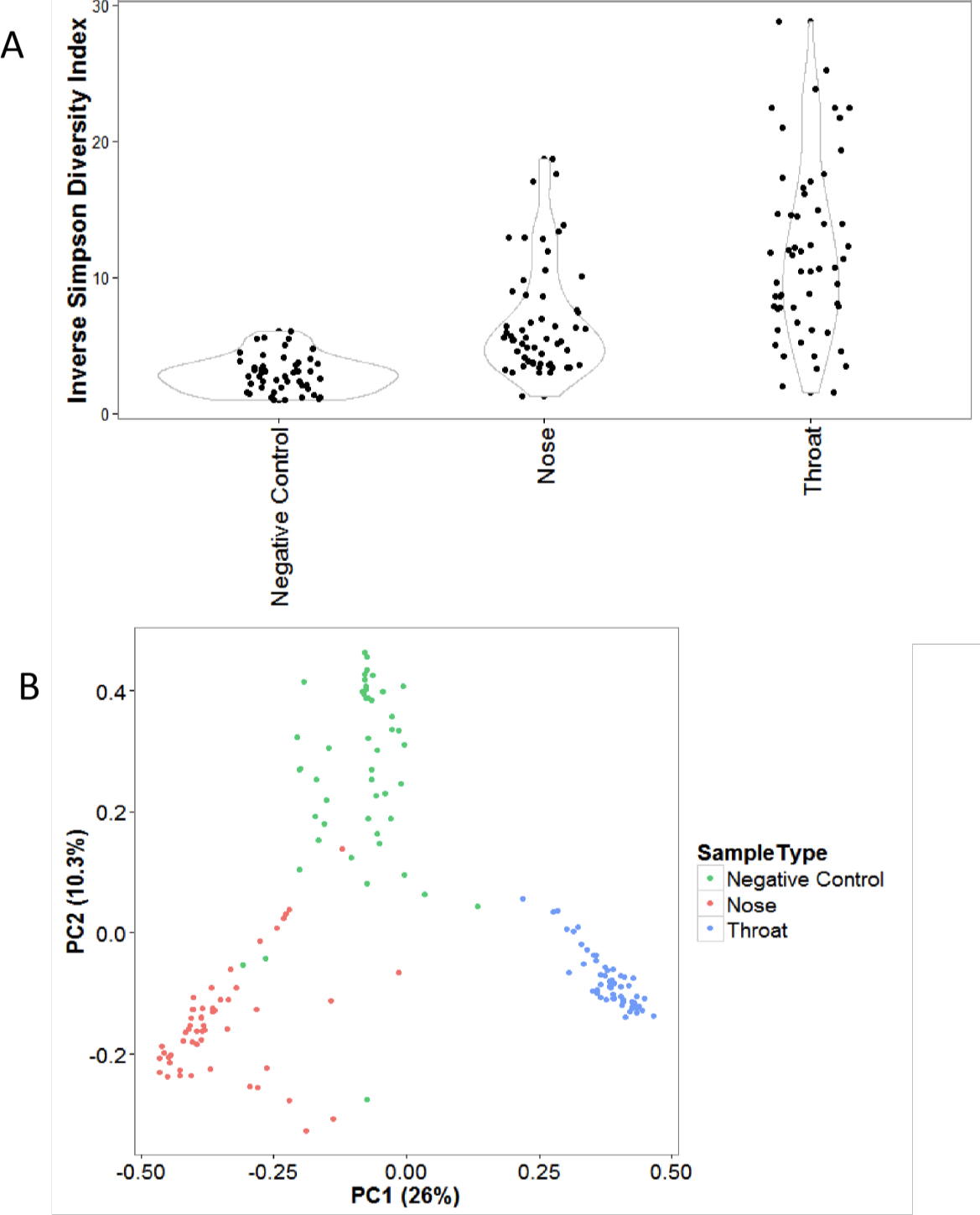


Figure S2-2: Diversity indexes for all samples. (a) Inverse Simpson diversity index violin plot. (b) Ordination plot of Bray-Curtis dissimilarly index of each samples microbiome. PC1 and PC2 = principal coordinates 1 and 2, respectively.


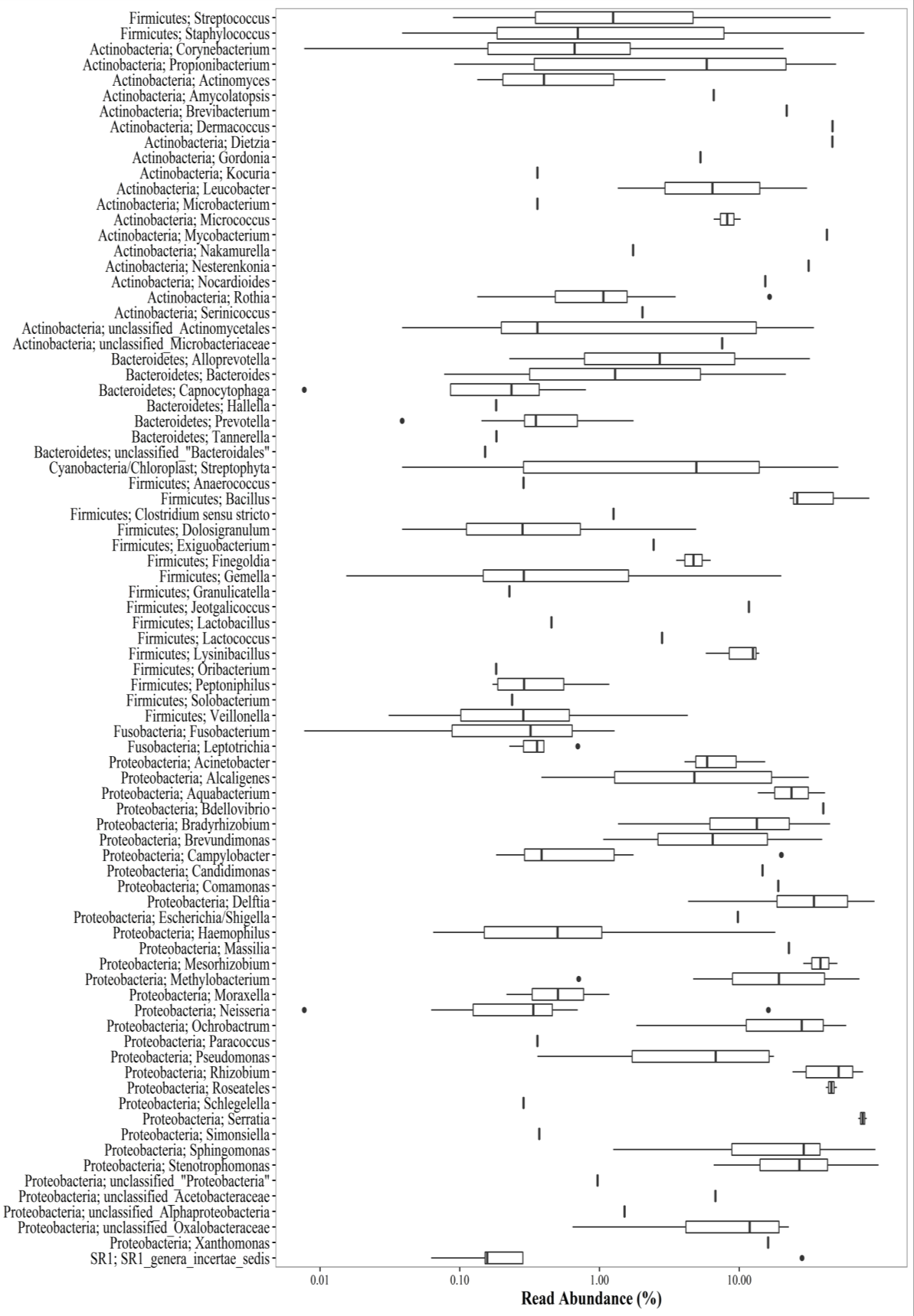


Figure S2-3: Boxplot of OTUs present in negative control samples (log scale)


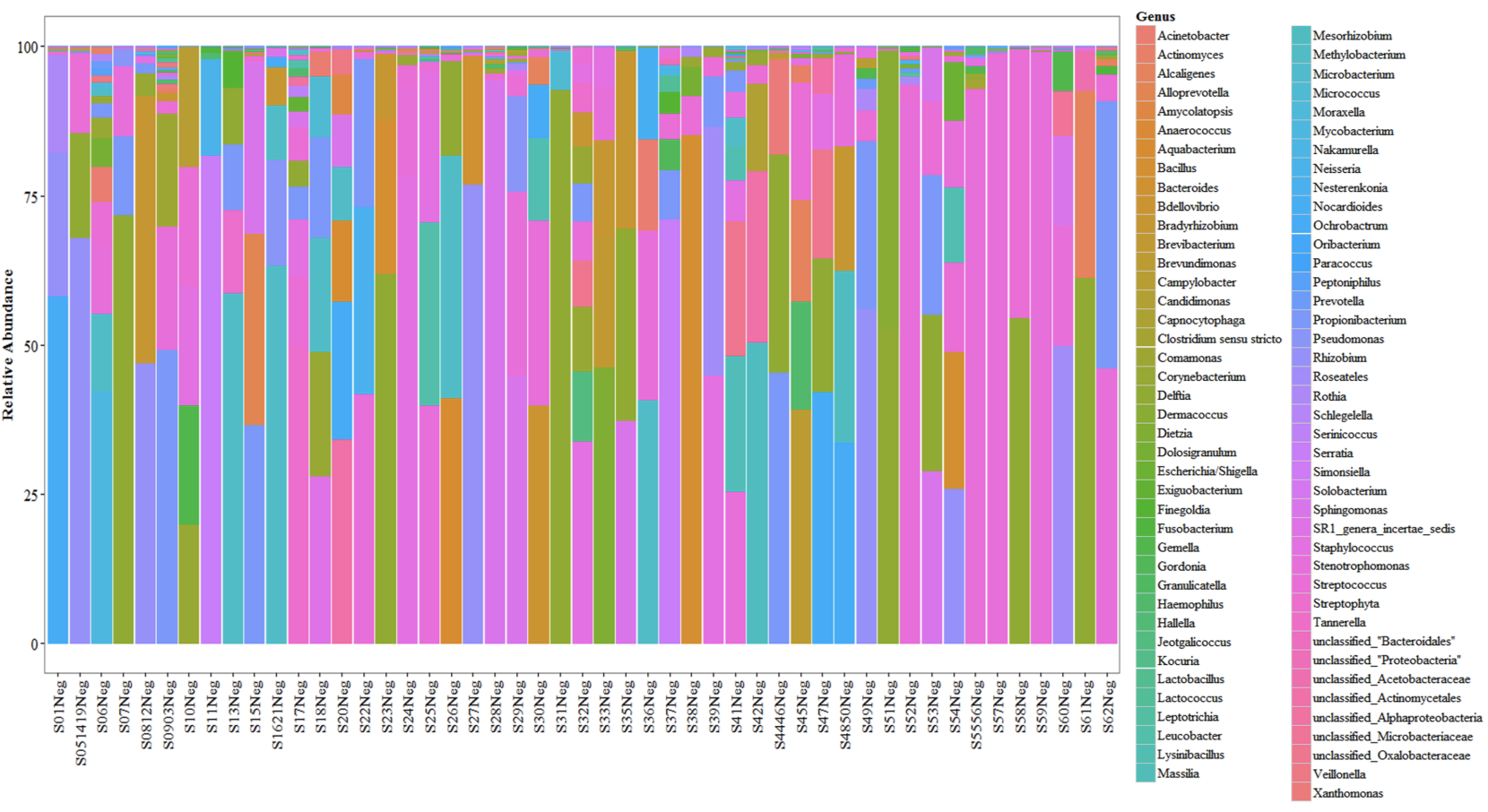


Figure S2-4: Barplot of relative abundances of OTUs present in the negative control samples.

1. Magoc T, Salzberg SL. FLASH: fast length adjustment of short reads to improve genome assemblies. Bioinformatics (Oxford, England). 2011;27(21):2957-63.

2. Martin M. Cutadapt removes adapter sequences from high-throughput sequencing reads. EMBnetjournal. 2011;17(1).

3. Edgar RC. UPARSE: highly accurate OTU sequences from microbial amplicon reads. Nat Meth. 2013;10(10):996-8.

4. Cole JR, Wang Q, Fish JA, Chai B, McGarrell DM, Sun Y, et al. Ribosomal Database Project: data and tools for high throughput rRNA analysis. Nucleic acids research. 2014;42(Database issue):D633-42.

5. Camacho C, Coulouris G, Avagyan V, Ma N, Papadopoulos J, Bealer K, et al. BLAST+: architecture and applications. BMC bioinformatics. 2009;10:421.
