# Supplementary material for: The nasal and oropharyngeal microbiomes of healthy livestock workers
Source: PLoS One. 2019 Mar 12;14(3):e0212949. doi: 10.1371/journal.pone.0212949 (PMC6413945; doi:10.1371/journal.pone.0212949)
Supplement: S3 File — This file contains table with additional output from the DESeq2 results. (DOCX) [file pone.0212949.s003.docx]

Table S6-1: Microbiota differentially abundant between those with and without livestock contact in the nares (full cohort)

| OTU | Log_2_-Fold Change | P-value | Adjusted p-value* | Phylum | Genus |
| --- | --- | --- | --- | --- | --- |
| OTU_534 | 24.76 | 3.36E-39 | 8.46E-37 | Cyanobacteria/Chloroplast | *Streptophyta* |
| OTU_27 | 9.80 | 4.46E-06 | 8.03E-05 | Proteobacteria | *Moraxella* |
| OTU_28 | 2.62 | 2.29E-04 | 2.31E-03 | Cyanobacteria/Chloroplast | *Streptophyta* |
| OTU_880 | -5.11 | 7.08E-04 | 6.87E-03 | Firmicutes | *Ruminococcus2* |
| OTU_610 | -5.35 | 1.48E-04 | 1.69E-03 | Firmicutes | *Blautia* |
| OTU_507 | -5.49 | 1.01E-04 | 1.49E-03 | Firmicutes | *Clostridium sensu stricto* |
| OTU_17 | -5.54 | 1.24E-04 | 1.64E-03 | Proteobacteria | *Moraxella* |
| OTU_1177 | -5.62 | 1.71E-04 | 1.88E-03 | Firmicutes | *Megasphaera* |
| OTU_2648 | -5.69 | 1.45E-04 | 1.69E-03 | Firmicutes | *Clostridium sensu stricto* |
| OTU_365 | -5.72 | 2.20E-04 | 2.31E-03 | Bacteroidetes | *Prevotella* |
| OTU_337 | -5.96 | 1.41E-04 | 1.69E-03 | Actinobacteria | *Rothia* |
| OTU_483 | -6.05 | 1.16E-04 | 1.62E-03 | Bacteroidetes | *Prevotella* |
| OTU_802 | -6.12 | 7.44E-07 | 1.56E-05 | Bacteroidetes | *Prevotella* |
| OTU_1058 | -6.29 | 2.23E-05 | 3.74E-04 | Firmicutes | *Oscillibacter* |
| OTU_291 | -6.33 | 3.97E-08 | 9.99E-07 | Firmicutes | *Aerococcus* |
| OTU_435 | -6.43 | 3.54E-05 | 5.58E-04 | Firmicutes | *Coprococcus* |
| OTU_282 | -6.60 | 1.39E-06 | 2.69E-05 | Firmicutes | *Lactobacillus* |
| OTU_209 | -6.84 | 7.82E-08 | 1.79E-06 | Firmicutes | *Clostridium XI* |
| OTU_108 | -7.43 | 2.49E-11 | 8.95E-10 | Firmicutes | *Clostridium sensu stricto* |
| OTU_747 | -7.64 | 8.46E-09 | 2.37E-07 | Bacteroidetes | *Prevotella* |
| OTU_130 | -8.28 | 1.88E-14 | 9.48E-13 | Firmicutes | *Lactobacillus* |
| OTU_40 | -8.30 | 2.40E-12 | 1.01E-10 | Firmicutes | *Turicibacter* |
| OTU_154 | -8.43 | 5.29E-11 | 1.67E-09 | Firmicutes | *Clostridium sensu stricto* |
| OTU_34 | -8.64 | 8.68E-15 | 5.47E-13 | Firmicutes | *Clostridium sensu stricto* |
| OTU_221 | -9.15 | 6.12E-17 | 7.71E-15 | Firmicutes | *Clostridium XI* |
| OTU_53 | -9.79 | 3.16E-16 | 2.65E-14 | Firmicutes | *Streptococcus* |

Table S6-2: Microbiota differentially abundant between those with and without livestock contact in the nares of males participants

| OTU | Log_2_-Fold Change | P-value | Adjusted p-value* | Phylum | Genus |  |
| --- | --- | --- | --- | --- | --- | --- |
| OTU_53 | -10.03 | 2.55E-15 | 6.11E-13 | Firmicutes | *Streptococcus* | |
| OTU_221 | -9.12 | 9.17E-14 | 1.10E-11 | Firmicutes | *Clostridium XI* | |
| OTU_34 | -9.18 | 3.42E-13 | 2.74E-11 | Firmicutes | *Clostridium sensu stricto* | |
| OTU_108 | -8.38 | 6.12E-12 | 3.67E-10 | Firmicutes | *Clostridium sensu stricto* | |
| OTU_130 | -8.90 | 9.92E-12 | 4.76E-10 | Firmicutes | *Lactobacillus* | |
| OTU_154 | -8.94 | 1.99E-10 | 7.96E-09 | Firmicutes | *Clostridium sensu stricto* | |
| OTU_40 | -7.82 | 2.07E-09 | 7.09E-08 | Firmicutes | *Turicibacter* | |
| OTU_747 | -7.87 | 4.32E-08 | 1.30E-06 | Bacteroidetes | *Prevotella* | |
| OTU_209 | -7.06 | 5.61E-08 | 1.50E-06 | Firmicutes | *Clostridium XI* | |
| OTU_291 | -6.75 | 9.95E-08 | 2.39E-06 | Firmicutes | *Aerococcus* | |
| OTU_802 | -6.42 | 4.10E-06 | 8.95E-05 | Bacteroidetes | *Prevotella* | |
| OTU_282 | -6.33 | 4.05E-05 | 8.11E-04 | Firmicutes | *Lactobacillus* | |
| OTU_1058 | -6.51 | 4.81E-05 | 8.88E-04 | Firmicutes | *Oscillibacter* | |
| OTU_435 | -6.68 | 7.17E-05 | 1.23E-03 | Firmicutes | *Coprococcus* | |
| OTU_17 | -5.67 | 1.25E-04 | 1.93E-03 | Proteobacteria | *Moraxella* | |
| OTU_507 | -5.73 | 1.29E-04 | 1.93E-03 | Firmicutes | *Clostridium sensu stricto* | |
| OTU_483 | -6.29 | 1.95E-04 | 2.75E-03 | Bacteroidetes | *Prevotella* | |
| OTU_1177 | -5.87 | 2.29E-04 | 2.90E-03 | Firmicutes | *Megasphaera* | |
| OTU_2648 | -5.89 | 2.23E-04 | 2.90E-03 | Firmicutes | *Clostridium sensu stricto* | |
| OTU_337 | -6.14 | 2.52E-04 | 3.02E-03 | Actinobacteria | *Rothia* | |
| OTU_365 | -5.96 | 2.86E-04 | 3.27E-03 | Bacteroidetes | *Prevotella* | |
| OTU_610 | -5.59 | 4.14E-04 | 4.52E-03 | Firmicutes | *Blautia* | |
| OTU_880 | -5.36 | 7.30E-04 | 7.62E-03 | Firmicutes | *Ruminococcus2* | |
| OTU_713 | -6.30 | 8.04E-04 | 8.04E-03 | Proteobacteria | *Psychrobacter* | |

Table S6-3: Microbiota differentially abundant between those with and without livestock contact in the nares of those over the age of 55

| OTU | Log_2_-Fold Change | P-value | Adjusted p-value* | Phylum | Genus |
| --- | --- | --- | --- | --- | --- |
| OTU_53 | -9.55 | 3.51E-11 | 6.49E-09 | Firmicutes | *Streptococcus* |
| OTU_34 | -8.36 | 7.52E-11 | 6.95E-09 | Firmicutes | *Clostridium sensu stricto* |
| OTU_221 | -9.00 | 1.80E-10 | 1.11E-08 | Firmicutes | *Clostridium XI* |
| OTU_130 | -8.39 | 6.53E-10 | 3.02E-08 | Firmicutes | *Lactobacillus* |
| OTU_108 | -6.72 | 6.21E-08 | 2.30E-06 | Firmicutes | *Clostridium sensu stricto* |
| OTU_40 | -7.67 | 9.73E-08 | 3.00E-06 | Firmicutes | *Turicibacter* |
| OTU_154 | -8.16 | 1.68E-07 | 4.32E-06 | Firmicutes | *Clostridium sensu stricto* |
| OTU_291 | -7.81 | 1.87E-07 | 4.32E-06 | Firmicutes | *Aerococcus* |
| OTU_802 | -7.64 | 6.36E-07 | 1.31E-05 | Bacteroidetes | *Prevotella* |
| OTU_747 | -7.68 | 1.64E-06 | 3.03E-05 | Bacteroidetes | *Prevotella* |
| OTU_209 | -5.81 | 2.94E-05 | 4.94E-04 | Firmicutes | *Clostridium XI* |
| OTU_7 | 3.72 | 7.64E-05 | 1.18E-03 | Firmicutes | *Streptococcus* |
| OTU_282 | -6.36 | 2.50E-04 | 3.56E-03 | Firmicutes | *Lactobacillus* |
| OTU_1058 | -6.19 | 4.15E-04 | 5.48E-03 | Firmicutes | *Oscillibacter* |
| OTU_713 | -6.38 | 5.61E-04 | 6.92E-03 | Proteobacteria | *Psychrobacter* |
| OTU_17 | -5.28 | 7.21E-04 | 8.34E-03 | Proteobacteria | *Moraxella* |

Table S6-4: Microbiota differentially abundant between those with and without livestock contact in the nares of those age 55 and under.

| OTU | Log_2_-Fold Change | P-value | Adjusted p-value* | Phylum | Genus |
| --- | --- | --- | --- | --- | --- |
| OTU_53 | -27.02 | 3.74E-19 | 4.60E-17 | Firmicutes | *Streptococcus* |
| OTU_27 | 23.94 | 8.79E-18 | 5.40E-16 | Proteobacteria | *Moraxella* |
| OTU_209 | -26.67 | 1.03E-16 | 4.22E-15 | Firmicutes | *Clostridium XI* |
| OTU_167 | 23.70 | 4.03E-13 | 1.24E-11 | Firmicutes | *Anaerococcus* |
| OTU_40 | -9.06 | 6.52E-05 | 1.60E-03 | Firmicutes | *Turicibacter* |
| OTU_221 | -8.69 | 1.26E-04 | 2.59E-03 | Firmicutes | *Clostridium XI* |
| OTU_130 | -8.41 | 2.12E-04 | 3.72E-03 | Firmicutes | *Lactobacillus* |
| OTU_54 | 3.99 | 5.78E-04 | 8.88E-03 | Proteobacteria | Unclassified *Proteobacteria* |

Table S6-5: Microbiota differentially abundant between livestock workers with and without swine contact in the oropharynx (n=12)

| OTU | Log_2_-Fold Change | P-value | Adjusted p-value* | Phylum | Genus |
| --- | --- | --- | --- | --- | --- |
| OTU_213 | 5.44 | 4.71E-06 | 4.25E-04 | Firmicutes | *Streptococcus* |
| OTU_23 | 5.24 | 5.29E-06 | 4.25E-04 | Proteobacteria | *Neisseria* |
| OTU_642 | 6.90 | 3.28E-05 | 1.76E-03 | Bacteroidetes | *Prevotella* |
| OTU_470 | 6.30 | 1.43E-04 | 4.60E-03 | Fusobacteria | *Leptotrichia* |
| OTU_5 | 3.39 | 1.19E-04 | 4.60E-03 | Fusobacteria | *Fusobacterium* |
| OTU_130 | -7.85 | 5.23E-04 | 1.40E-02 | Firmicutes | *Lactobacillus* |
| OTU_35 | 5.45 | 6.53E-04 | 1.50E-02 | Proteobacteria | *Haemophilus* |
| OTU_1250 | -7.06 | 1.82E-03 | 3.25E-02 | Bacilli | *Lactobacillus* |
| OTU_201 | 5.49 | 1.65E-03 | 3.25E-02 | Bacteroidetes | *Capnocytophaga* |
| OTU_2524 | -6.98 | 2.03E-03 | 3.26E-02 | Firmicutes | *Lactobacillus* |
| OTU_251 | 5.29 | 2.33E-03 | 3.42E-02 | Bacteroidetes | *Alloprevotella* |
| OTU_294 | 5.14 | 2.91E-03 | 3.91E-02 | Firmicutes | *Peptostrepotococcus* |
